# Eco-ISEA3H, a machine learning ready spatial database for ecometric and species distribution modeling

**DOI:** 10.1038/s41597-023-01966-x

**Published:** 2023-02-07

**Authors:** Michael F. Mechenich, Indrė Žliobaitė

**Affiliations:** 1 Department of Computer Science, University of Helsinki, 00014 Helsinki, Finland; 2 Department of Geosciences and Geography, University of Helsinki, 00014 Helsinki, Finland

**Keywords:** Biogeography, Evolutionary ecology

## Abstract

We present the Eco-ISEA3H database, a compilation of global spatial data characterizing climate, geology, land cover, physical and human geography, and the geographic ranges of nearly 900 large mammalian species. The data are tailored for machine learning (ML)-based ecological modeling, and are intended primarily for continental- to global-scale ecometric and species distribution modeling. Such models are trained on present-day data and applied to the geologic past, or to future scenarios of climatic and environmental change. Model training requires integrated global datasets, describing species’ occurrence and environment via consistent observational units. The Eco-ISEA3H database incorporates data from 17 sources, and includes 3,033 variables. The database is built on the Icosahedral Snyder Equal Area (ISEA) aperture 3 hexagonal (3H) discrete global grid system (DGGS), which partitions the Earth’s surface into equal-area hexagonal cells. Source data were incorporated at six nested ISEA3H resolutions, using scripts developed and made available here. We demonstrate the utility of the database in a case study analyzing the bioclimatic envelopes of ten large, widely distributed mammalian species.

## Background & Summary

Human activity is rapidly altering the Earth system^1^, with grave consequences for biotic and human communities^2^. In the Anthropocene epoch, it is increasingly important to understand the complex interdependencies of environment and species distributions, and to predict ecosystem response to changing conditions. In this context, we increasingly look to assess and describe the condition of the global Earth system, and to model the relationships among system components in normal as well as exceptional circumstances.

Ecometric modeling^3–5^ quantifies relationships between the functional traits of communities of organisms and their environments. This quantitative modeling approach relies on understanding certain phenotypic traits as an adaptive response to environmental conditions, an understanding also used qualitatively to reconstruct past climates^6,7^. Classical statistical and state-of-the-art machine learning techniques are commonly utilized. Ecometric models are trained on present species distribution and environmental datasets; studies variably focus on prediction in the present^8–13^, or on quantitatively reconstructing climatic and environmental conditions of the past^14–18^, including the context of early human evolution^19,20^. However, in all cases ecometric models are intended to be transferable to the geologic past, by utilizing traits which persist in faunal communities for 20 to 30 million years, and which preserve in the fossil record.

Methods of ecometric modeling closely relate to those of species distribution modeling (SDM)^21–24^, also known as environmental or ecological niche modeling. Species distribution modeling is a general term referring to computational modeling for quantifying associations between organisms and their environments. Classical species distribution modeling aims at predicting the probability of species occurrence as a function of environmental conditions. While species distribution modeling has traditionally focused on local or regional scales, analyses at increasingly large scales are gaining popularity. Machine learning methods are becoming the leading tools for such analyses^25–28^.

Developing ecometric and species distribution models requires similar large-scale, integrated datasets, describing species’ occurrences and traits, and the environmental context of those occurrences. The results of many global mapping efforts are already available; examples include the Map of Life^29^, describing species’ geographic distributions, and WorldClim^30,31^, describing climatic averages and extremes. These datasets provide essential variables for monitoring changes in climate^32^ and biodiversity^33^. However, spatial datasets are often published in differing coordinate reference systems, spatial resolutions, geographic data models, and file formats. Before proceeding to ecometric or species distribution modeling, an interested researcher must invest considerable effort in integrating these datasets, mapping them to shared spatial units of analysis. Many ecometric and SDM studies require the same global datasets and the same data preprocessing, even if the ultimate modeling goals vary considerably.

Our aim was to develop a unified spatial database, built on consistent spatial units of observation and analysis, which may be used directly in continental- to global-scale ecometric and species distribution modeling. To this end, we utilized the Icosahedral Snyder Equal Area (ISEA) aperture 3 hexagonal (3H) discrete global grid system (DGGS)^34^. A DGGS is a hierarchical system by which the Earth’s surface is divided into observational units: it is *discrete*, in that it discretizes the surface into areal cells; it is *global* in spatial scope; and it is a *grid system*, in that it defines regular grids of cells at a number of spatial resolutions^35^. Finally, it is *hierarchical*, in that a systematic relationship exists between grid cells at one resolution, and those at the next coarser or finer resolution.

DGGSs are an essential component of the Digital Earth (DE) vision; such systems provide a regular, systematic spatial framework with which we may integrate the rapidly growing, multiple-source compendium of geospatial data available today^36^. We selected the ISEA3H DGGS because it partitions the Earth’s surface into regular grids of equal-area hexagonal cells. These observational units have uniform topology with neighbors, each sharing an edge with six adjacent cells, and are maximally compact, minimizing within-unit variability in expectation.

The resulting Eco-ISEA3H database^37^ includes the geographic distributions of extant and recently extinct large mammalian species in the orders Artiodactyla, Perissodactyla, Primates, and Proboscidea, as well as the environmental context of their presence and absence, characterized by climate, geology, land cover, and physical and human geography. Source datasets are sampled and summarized by the hexagonal cells of the ISEA3H DGGS, at six nested levels of resolution.

We intended this to be a resource for students and researchers in the life and computational sciences, to be used without advanced knowledge of geospatial data processing required. Component datasets are preprocessed and provided in a plain-text, tabular format, allowing interested researchers to focus attention on computational analysis and modeling. The database may also be used as a benchmark dataset for systematic comparison of differing computational modeling approaches. Finally, we include scripts for mapping source spatial datasets to the ISEA3H grid system.

## Methods

Our objective in developing the Eco-ISEA3H database^37^ was to compile a coordinated, global set of tabular data, characterizing environmental conditions and the geographic distributions of large mammalian species. The database was built on the ISEA3H DGGS, a multi-resolution system of global grids, each grid dividing the Earth’s surface into discrete, equal-area hexagonal cells. These cells constitute areal *units of observation*, uniformly resampling data provided in different coordinate reference systems, spatial resolutions, geographic data models, and file formats. We included data at six consecutive ISEA3H resolutions, in which cell centroid spacing ranges from 29 kilometers to approximately 450 kilometers.

Eco-ISEA3H themes and variables were derived from 17 geospatial data sources, and represent 3,033 features to be used for ML-based predictive modeling. Source datasets were published in *raster* or *vector* format, data models built on fundamentally different representations of spatial phenomena. Raster datasets comprise regular arrays of pixels, each pixel holding a value, while vector datasets comprise point, line, and polygon features, each feature defined by one or more (*x, y*) coordinate pairs and attributed with one or more values. Our task was to integrate these disparate source datasets, resampling and summarizing the values of raster pixels and vector features via the discrete, equal-area cells of the ISEA3H global grid system. The hexagonal cells on which the Eco-ISEA3H database^37^ is built thus serve as unifying observational units for SDM and ecometric analysis and modeling.

From the statistical and ML perspective, each areal observational unit is characterized by (1) a set of environmental variables, representing climatic conditions, soil and near-surface lithology, land cover, and physical geography; and (2) a set of occurrence variables, representing the present and estimated natural distributions of large mammalian species. Predictive modeling tasks for statistical and ML modeling can be formulated in two directions: predicting species’ occurrences as a function of climatic and other environmental conditions (as in SDM studies), or predicting climatic and other environmental conditions as a function of species’ occurrences and functional traits (as in ecometric studies).

### Spatial units of observation

To study continuous spatial phenomena over a region of interest, it is often necessary to divide the region into a number of discrete, areal observational units, which may be used in statistical summaries and/or modeling. Machine learning methods for ecometric and species distribution modeling require discrete observational units, each characterized by two sets of variables, one describing environmental conditions, the other species’ geographic distributions. A major question in data representation concerns the form of these units; defining discrete spatial units of observation constitutes a well-known problem in geography, termed the modifiable areal unit problem (MAUP)^38^. As we change the size of proposed observational units, or change the boundaries between units while holding unit areas constant, measures of interest within these units - and derived summary statistics and model parameters - may differ; these are termed the “scale” and “zone” effects, respectively^38^.

Our objective in utilizing the ISEA3H DGGS^34^ was to implement a robust spatial division of the Earth’s surface. The grid cells of the DGGS *discretize* the Earth’s sphere, forming, at each DGGS resolution, a global set of areal observational units with which to sample and summarize source datasets. To be optimally effective in the observation, simulation, and visualization of spatial phenomena, such a grid must meet certain structural criteria. We propose, modifying the *Goodchild Criteria*^39^, the DGGS grid must contain (1) contiguous, (2) equivalent observational units, (3) minimizing intra-unit variability, (4) having uniform topology with neighboring units, and (5) being visually effective, facilitating interpretation and communication. Each criterion will be discussed in detail; further, we will argue the ISEA3H DGGS selected for this study satisfies these criteria.

#### Contiguity & congruency

We suggest that a *regular tiling* maximally satisfies the criteria of (1) contiguity and (2) equivalence. A *tiling* is simply a set of shapes which cover a plane without gaps or overlaps^40^. A regular tiling is one of a class of tilings in which the tiles - our observational units - are highly equal; such tilings are monohedral, and composed of congruent, regular (equiangular and equilateral) polygons. Thus, regular tilings are also highly symmetrical, being vertex-, edge-, tile-, and flag-transitive. Three regular polygons may be used to create a regular tiling: the equilateral triangle, the square, and the regular hexagon^40^.

With this suggestion, we follow common convention; in ecology, grids of square (or rectangular) cells are most often utilized, motivated in part by the use of raster datasets^41^, made of rectilinear rows and columns of pixels. However, it should be noted that while the square cells of these grids are equal in the coordinate reference system in which they are defined, such cells are rarely congruent, or indeed even square, on the Earth’s surface. The properties of the ISEA projection selected for this DGGS - area preservation, and relatively low angular distortion - serve to retain considerable congruency when inversely projecting grid cells to the spherical surface of the Earth.

#### Compactness

To accurately represent the spatially continuous phenomena of the Earth system, the grid cells of a DGGS - the areal observational units used in summarizing, modeling, and visualizing - must effectively discretize these phenomena. Thus, the DGGS must be structured such that (3) intra-unit variability is minimized, and inter-unit variability is maximized. In this way, patterns of variation among units more accurately represent patterns of variation inherent in the phenomena.

Intra-unit variability may be minimized, in expectation, by *compact* observational units. Tobler’s oft-cited first law of geography serves as explanation: “everything is related to everything else, but near things are more related than distant things”^42^. Thus, compact units, in which all portions of the interior are nearer each other, are expected to contain less interior variability than elongated units, in which portions of the interior may be more distant. Given these properties, compact units are optimal in the context of DGGS development, elongated units in the context of efficient ecological sampling.

Regular hexagons are the most compact of the three polygons - the equilateral triangle, square, and regular hexagon - admitting regular tilings. This compactness may be expressed in several related and complementary ways. First, of any equal-area tiling, regular hexagons have the minimum possible ratio of perimeter to area^43^. In minimizing perimeter length per unit area, regular hexagons are thus the most circle-like of the polygons admitting equal-area tilings. Relatedly, regular hexagonal packing is the highest-density arrangement of equal-area circles on a plane^44^.

Finally, a regular hexagonal lattice optimally *quantizes* a plane; of the polygons admitting regular tilings, regular hexagons minimize the mean squared distance of any point to the nearest polygon centroid^45^. This distance, or “dimensionless second moment,” quantifies the more qualitative notion of interior nearness discussed in relation to Tobler’s Law.

#### Topology

In addition to maximally satisfying the (3) compactness criterion, regular hexagons have a topological advantage over equilateral triangles and squares. Of these three regular polygons, hexagons have the simplest relationship with neighbors in a tiling or grid, each (4) uniformly sharing an edge with the six adjacent hexagons forming its first-order neighborhood. Triangles and squares, in contrast, share only a single vertex with three or four neighbors, respectively, and an edge with three or four neighbors, complicating the definition of neighborhood in these grids.

It follows that hexagonal topology has greater angular resolution than edge-based triangular or square topologies; movement may be simulated between cells in six directions, rather than in three or four, respectively. These properties - neighborhood simplicity and angular resolution - were confirmed by Golay^46^, in the context of pattern transformation operations on two-dimensional arrays. Further, these properties likely account for the widespread use of hexagonal grids in strategy board games, since these grids were introduced in the early 1960s^47^.

Differing grid topologies affect the results of ecological models simulating dispersal. White and Kiester^48^, for example, found the topology of the network of communities in a neutral community ecology model - in which simulated communities had hexagonal neighborhoods, or von Neumann, Moore, or Margolus neighborhoods - affected modeled species abundances and diversities, but in complex ways, which differed given different model parameter values. (Note that the four neighbors with which a square cell shares an edge are termed its *rook*, or *von Neumann* neighborhood, and these plus the four neighbors with which it shares a single vertex its *queen*, or *Moore* neighborhood.)

#### Visualization

Finally, in addition to these gains in representational accuracy, (5) hexagonal tilings are more visually effective than square tilings. Whether used in cartography or other two-dimensional data visualization, tilings inevitably create visual lines, artifacts of the lattice of shared edges between tiles^49^. Given our “sense of gravitational balance,” Carr *et al*.^49^ argue the horizontal and vertical lines of square tilings strongly distract the human eye, obscuring data-driven patterns in a dataset so visualized. The non-orthogonal lines of hexagonal tilings, however, feature less prominently, and thus distract less from patterns of interest^49^.

Note that this is not an issue of aesthetics only: maps are often essential tools in scientific reasoning and communication, and effective visualization is important. Indeed, Carr *et al*.^49^ suggest this visual advantage makes a stronger case for hexagonal tilings than the representational advantages discussed previously.

### DGGS sampling workflows

**Fig. 1:**
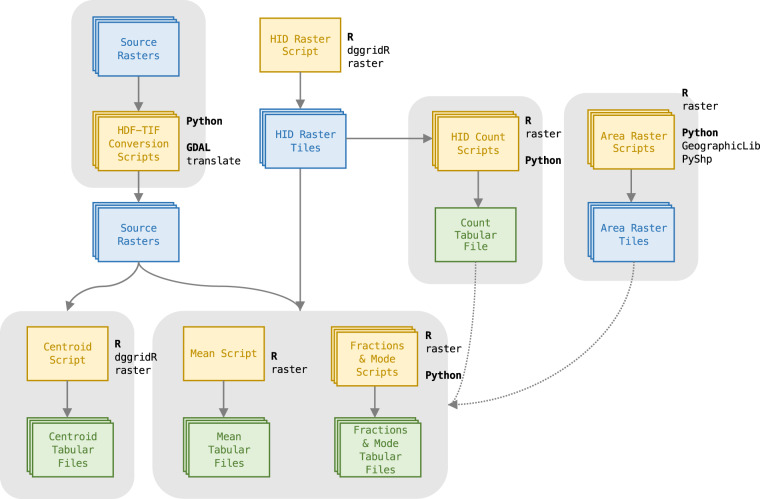
Workflow developed to incorporate raster datasets into the ISEA3H DGGS.

**Fig. 2:**
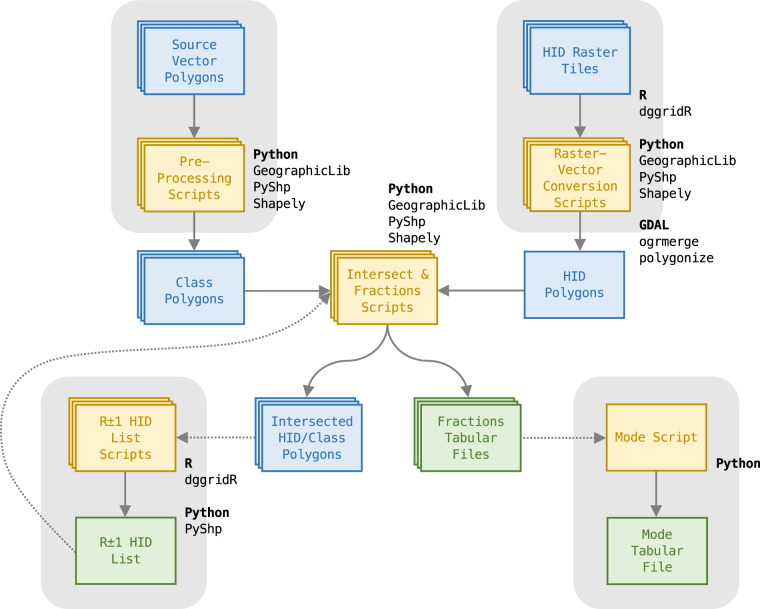
Workflow developed to incorporate vector datasets into the ISEA3H DGGS.

The set of scripted workflows developed to incorporate spatial datasets into the Eco-ISEA3H database^37^ utilize published spatial libraries and packages for Python and R, and include several validation steps, intended to verify the integrity of source datasets and the fidelity of the transfer to the DGGS. Workflows developed for raster datasets are presented in Fig. 1, and workflows for vector datasets in Fig. 2.

To begin, one general principle guides each workflow: *each source dataset is processed in its native coordinate reference system*. In all cases, a representation of the DGGS is developed in the coordinate reference system of the source dataset, and used in summarizing that dataset. The guiding premise here is that the spatial dataset is as the authors intended it in the coordinate reference system in which it is published and distributed.

This is especially relevant for vector polygon datasets. Consider, for example, certain species’ range polygons published by the IUCN Red List^50^; these polygons are defined only roughly, having relatively few, widely spaced vertices, connected by arcs many hundreds of kilometers in length. These arcs are “straight” in the plate carrée projection with which the dataset’s WGS84 latitude/longitude coordinates are visualized by default. If vertex coordinates were projected into another coordinate reference system, the arcs would be similarly “straight” in this new system, and thus potentially trace very different paths across the Earth’s surface. Absent information to the contrary, we assume the arcs are as intended in the reference system in which the data are distributed.

The spatial structure of raster datasets depends similarly on each dataset’s coordinate reference system; rasters are made of rows and columns of pixels, rectilinear and orthogonal only in the raster’s native coordinate reference system. We assume raster pixels are “atomic” units, each indivisible and representative of the area it natively covers. Thus, we query the DGGS at each pixel’s centroid, and assign the pixel wholly to the coincident DGGS cell.

### Raster dataset processing

If necessary, source raster datasets were first converted to the GeoTIFF file format, so that the files were readable in the open-source GIS software used later in the processing workflow. GeoTIFF files are simply Tag Image File Format (TIFF) image files with embedded georeferencing information, describing the dataset’s spatial extent and coordinate reference system. Hierarchical Data Format Release 4 (HDF4) files were converted to GeoTIFF format using the Geospatial Data Abstraction Library (GDAL) *translate* utility^51^.

Next, raster tiles containing ISEA3H hexagon identification (HID) indexing numbers were generated; these integer HIDs uniquely identify each cell at a given ISEA3H resolution. A set of HID raster tiles was required for each source raster dataset, for each ISEA3H resolution, because (1) GeoTIFF rasters are able to hold only a single value at each pixel; and (2) HIDs sequentially number cells at a given ISEA3H resolution, from 1 to the number of cells present at that resolution. Thus, HIDs are not unique between resolutions; HID 84, for example, identifies a cell at each ISEA3H resolution 2 and higher.

The HID raster tiles generated for a source raster dataset matched that dataset’s grid resolution, extent, and coordinate reference system precisely; thus, there was a one-to-one correlation between the pixels of the HID raster tiles and the source raster dataset tiles. For each tile, pixel centroid coordinates were passed to the *dggridR* package^52^ for R, which returned the ISEA3H cell identification number for that location. In this way, the pixels of the source raster were treated as indivisible units, assigned wholly to a particular HID on the basis of each pixel’s centroid. HID rasters were written in GeoTIFF format using the *raster* package^53^ for R.

In equal-area projected coordinate reference systems, simple counts of the number of raster pixels assigned to each HID were sufficient to determine each ISEA3H cell’s total area. In all other cases - for example, for raster datasets using the World Geodetic System 1984 (WGS84) coordinate reference system - raster tiles containing pixel areas were generated. These areas were calculated by passing each pixel’s corner coordinates to the GeographicLib library^54^ for Python.

Finally, source raster dataset tiles, HID raster tiles, and area raster tiles (for source rasters using non-authalic coordinate reference systems) were superimposed to generate summary tabular files, describing the features of the source raster dataset by ISEA3H cell. The specifics of this process, which utilized functions of the *raster* package^53^ for R, depended on whether the source raster contained discrete, categorical values, or continuous, real-numbered values.

#### Discrete themes

For each source raster dataset containing discrete pixel values, one or more of the following summary statistics were calculated. While the *centroid* attribute requires a simple point sample, the *fraction* and *mode* attributes are area-integrated, and involve a multiple-step sampling process. For rasters using an authalic coordinate reference system, the *raster* package’s *crosstab* function^53^ was used to generate a contingency table for each tile; applied to source raster and HID raster tiles, the function tallied the number of pixels of each class coincident with each HID, for each tile. These tile-specific tables were then summed, to obtain total counts of pixels of each class within each HID.

For rasters using a non-authalic coordinate reference system, area raster tiles were required as well. For each tile, a vector of classes present in the source raster was assembled. For each of these classes in turn, a mask raster tile was generated, retaining pixels belonging to the class, and screening pixels belonging to all other classes. This mask was applied to the area raster tile, and retained pixels were summed within each HID using the *raster* package’s *zonal* function^53^. Thus, a contingency table was compiled for each raster tile, containing the area of each class within each HID. Finally, these tile-specific tables were summed, to obtain the total area of each class within each HID.

- **Centroid.** The *centroid* attribute records the categorical value occurring at each ISEA3H cell’s centroid. Where the source raster dataset contains a *null* value at a centroid, the cell is assigned a flag signifying no value is available.
- **Fraction.** The *fraction* attributes record the proportion of each ISEA3H cell’s area covered by each categorical value. For example, the Köppen-Geiger climate classification system, as implemented by Beck *et al*.^55^, includes 30 classes, listed in Table 4. Thus, each ISEA3H cell has an associated set of 30 *fraction* attributes for this dataset, recording the proportions of the cell’s area covered by the 30 categorical values, from tropical rainforest (Af) to polar tundra (ET).
- **Mode.** The *mode* attribute records the categorical value covering the greatest proportion of each ISEA3H cell’s area. For example, if an ISEA3H cell had a *fraction* value of 0.4 for some hypothetical categorical value *A*, 0.3 for *B*, and 0.3 for *C*, it would be assigned a *mode* value of *A*. A *mode* attribute is specified for cells in which the sum of the *fraction* attributes is greater than or equal to 0.2; where *fraction* attributes total less than 0.2, a flag signifying no value is assigned.

#### Continuous variables

For each source raster dataset containing continuous pixel values, one or more of the following summary statistics were calculated. Again, the *centroid* attribute requires only a simple point sample, while the *mean* attribute is area-integrated, requiring area raster tiles for source rasters using a non-authalic coordinate reference system.

- **Centroid.** The *centroid* attribute records the continuous value occurring at each ISEA3H cell’s centroid. Where the source raster dataset contains a *null* value at a centroid, the cell is assigned a flag signifying no value is available.
- **Mean.** The *mean* attribute records the area-weighted arithmetic mean of the continuous values of raster pixels within each ISEA3H cell. For raster datasets in authalic coordinate reference systems, the area-weighted mean is equivalent to the simple mean of the values of raster pixels within each cell; however, in all other cases, pixel values are weighted by pixel areas per the equation below, in which *w*_*i*_ and *x*_*i*_ indicate the area and value, respectively, of each pixel *i* within an ISEA3H cell containing *n* pixels.

$$\overline{x}=\frac{{\sum }_{i=1}^{n}{w}_{i}{x}_{i}}{{\sum }_{i=1}^{n}{w}_{i}}$$

For each tile, source raster values and area values were multiplied, pixel by pixel, using the *raster* package’s * arithmetic operator^53^. The resulting product raster tile, as well as the area raster tile, were then summed within each HID using the *raster* package’s *zonal* function^53^. Finally, these tile-specific tables were summed, to obtain both the numerator (summed product values) and denominator (summed area values) for the above equation, for each HID.

### Vector dataset processing

Source vector datasets incorporated into the Eco-ISEA3H database^37^ contain *polygon* features, discrete areas assigned a categorical value. A dataset may (1) contain polygons of several different classes; for example, the vector *shapefile* published by Olson *et al*.^56^ contains ecoregion polygons, each assigned to one of several biogeographic realms. Alternatively, a dataset may (2) represent a single class, with polygons indicating class presence; for example, the *shapefiles* published by the IUCN Red List^50^ each represent a species’ geographic range, with polygons indicating regions the species is present. In both cases, the summary statistics discussed in reference to raster datasets containing discrete values may be calculated.

Prior to inclusion in the Eco-ISEA3H database^37^, source vector datasets were preprocessed. To simplify the geographic representation of the class(es) of interest - that is, to remove unnecessary polygon boundaries - dataset polygons were dissolved, either on the class attribute in case (1), or globally in case (2), using the QGIS open-source desktop GIS application. The geodesic areas of dissolved polygons were then calculated using the GeographicLib library^54^. Finally, the geometries of dissolved polygons were checked for conformance with the OGC *Simple Feature Access* standard^57^ using the Shapely library^58^ for Python, ensuring these features served as valid input in the processing workflow to follow.

The intersection of source dataset polygons and ISEA3H cell polygons is central to the vector processing workflow. Source polygons result from the preliminary simplification and verification steps just discussed; cell polygons result from *polygonizing* a set of HID raster tiles for the ISEA3H resolution of interest. The polygonizing procedure utilized the open-source GDAL command-line tools *polygonize* and *ogrmerge*^51^, as well as the GeographicLib^54^ and Shapely^58^ libraries. Polygonizing HID raster tiles of the appropriate coordinate reference system (specifically, the system matching that of the source polygon dataset) ensured HID polygon boundaries displayed both proper geodesic curvature and the shape distortion induced by the ISEA map projection.

*Intersection* is a set-theoretic operation, returning polygons representing each coincident class/HID combination. The operation was implemented via the Shapely library^58^, and the geodesic areas of intersected polygons were calculated via the GeographicLib library^54^. Note that the scripted intersection tools developed for the Eco-ISEA3H database^37^ allow limiting the ISEA3H cells included in a single tool run, to break the processing of large datasets into manageable pieces. Runs may be limited to a user-specified range of HIDs. Additionally, if cells at the next coarser or finer ISEA3H resolution have been intersected with the source dataset, cells retained by the operation may be used as a spatial index; a list of coincident HIDs at the ISEA3H resolution of interest may be generated, and used to limit tool runs.

An output *shapefile* is written, containing intersected polygons attributed with the geodesic area, the HID, and in case (1), the source class. Next, an additional verification of the geometries of these intersected polygons is performed. Each intersected polygon is superimposed over the original ISEA3H cell polygon having the same HID. If intersected polygons have too few vertices to be valid, or are not contained by the original cell polygon from which each was derived, these polygons are flagged for review and revision. This step was implemented to catch geometry errors observed early in the development of the Eco-ISEA3H intersection tools.

Finally, the geodesic areas of intersected polygons are totaled, and the total area of each class within each HID is calculated. Dividing by the geodesic areas of the original ISEA3H cell polygons, these class totals are expressed as fractions of each cell’s total area. In two final verification steps, (1) the total intersected area of each class, across all HIDs, is compared to the area of the same class in the source dataset; and (2) class fraction values are confirmed to be less than or equal to unity within each HID. Deviations are flagged for review and revision.

### Data sources & themes

The Eco-ISEA3H database^37^ incorporates 17 source datasets, characterizing the Earth’s climate, geology, land cover, and physical geography, as well as human population density and the geographic ranges of nearly 900 large mammalian species. Data sources are listed in Table 1. We first present a brief overview of these sources, and describe sources and themes in greater detail in the following sections.

**Table 1:** Source datasets and themes included in the Eco-ISEA3H database^37^. Each dataset is described by full and abbreviated name, source, spatial resolution (for datasets published/distributed at more than one resolution), version, and scenario. Each theme is described by full and abbreviated name and type (whether it contains discrete, categorical values or continuous, real-valued variables).

Dataset	Source	Spatial Resolution	Version	Scenario	Theme	Theme Type
**Climate**						
Community Climate System Model Version 4 (CCSM4)	Sillmann *et al*.^61^,^62^	.	.	Historical	Expert Team on Climate Change Detection & Indices (ETCCDI) Climate Extremes Indices	Continuous
	Hijmans *et al*.^30^	.	.	RCP 8.5	Bioclimatic Variables (BIO)	Continuous
European Centre for Medium-Range Weather Forecasts (ECMWF)	Sillmann *et al*.^61,62^	.	ERA-40	.	Expert Team on Climate Change Detection & Indices (ETCCDI) Climate Extremes Indices	Continuous
ENVIronmental Rasters for Ecological Modeling (ENVIREM)	Title & Bemmels^60^	30 Arc-Second	1.0	.	Climatic Variables	Continuous
GLOH2O	Beck *et al*.^55^	.	1.0	.	Köppen-Geiger Climate Classification	Discrete
WorldClim	Hijmans *et al*.^30^	30 Arc-Second	1.4	.	Bioclimatic Variables (BIO)	Continuous
	Fick & Hijmans^31^	30 Arc-Second	2.0	.	Bioclimatic Variables (BIO)	Continuous
					Monthly Precipitation (PREC)	Continuous
					Monthly Solar Radiation (SRAD)	Continuous
					Monthly Mean Temperature (TAVG)	Continuous
					Monthly Minimum Temperature (TMIN)	Continuous
					Monthly Maximum Temperature (TMAX)	Continuous
					Monthly Vapor Pressure (VAPR)	Continuous
					Monthly Wind Speed (WIND)	Continuous
**Geology**						
Digital Soil Map of the World (DSMW)	Food & Agriculture Organization (FAO)^65^	.	3.6	.	Soil Units	Discrete
Global Lithological Map (GLiM)	Hartmann & Moosdorf^66^	.	1.1	.	Lithology	Discrete
Sedimentary Basins	Nyberg & Howell^67^	.	.	.	Structure	Discrete
**Human Geography**						
Gridded Population of the World (GPW)	Center for International Earth Science Information Network (CIESIN)^68^	30 Arc-Second	4.11	.	Population Density	Continuous
**Land Cover**						
MCD12Q1	Friedl & Sulla-Menashe^69^	.	6.0	.	International Geosphere-Biosphere Programme (IGBP) Land Cover Classification	Discrete
MOD44B	DiMiceli *et al*.^70^	.	6.0	.	Vegetation Continuous Fields (VCF)	Continuous
**Physical Geography**						
ENVIronmental Rasters for Ecological Modeling (ENVIREM)	Title & Bemmels^60^	30 Arc-Second	1.0	.	Topographic Variables	Continuous
Natural Earth (NE)	.	1:10 M	4.1.0	.	Lakes	Discrete
					Land	Discrete
					Minor Islands	Discrete
					Terra	Discrete
SRTM30-PLUS	Becker *et al*.^72^	.	11	.	Elevation	Continuous
World Wildlife Fund (WWF) Terrestrial Ecoregions (TE)	Olson *et al*.^56^	.	2.0	.	Biogeographic Realms	Discrete
World Wildlife Fund (WWF) Global Lakes & Wetlands Database (GLWD)	Lehner & Döll^71^	.	1.0	.	Level 3	Discrete
**Species Ranges**						
Red List (RL) of Threatened Species	International Union for Conservation of Nature (IUCN)^50^	.	2019.1	.	Artiodactyla: Antilocapridae, Bovidae, Camelidae, Cervidae, Giraffidae, Hippopotamidae, Moschidae, Suidae, Tayassuidae, Tragulidae	Discrete
					Perissodactyla: Equidae, Rhinocerotidae, Tapiridae	Discrete
					Primates: Aotidae, Atelidae, Callitrichidae, Cebidae, Cercopithecidae, Cheirogaleidae, Daubentoniidae, Galagidae, Hominidae, Hylobatidae, Indriidae, Lemuridae, Lepilemuridae, Lorisidae, Pitheciidae, Tarsiidae	Discrete
					Proboscidea: Elephantidae	Discrete
Phylogenetic Atlas of Mammal Macroecology (PHYLACINE)	Faurby *et al*.^73,74^	.	1.2.1	Present	Artiodactyla	Discrete
					Perissodactyla	Discrete
					Primates	Discrete
					Proboscidea	Discrete
				Present Natural	Artiodactyla	Discrete
					Perissodactyla	Discrete
					Primates	Discrete
					Proboscidea	Discrete

Climate is characterized primarily by temperature- and precipitation-based averages and extremes, summarized over the past 50 to 70 years, and forecasted for 40 to 60 years in the future under the RCP 8.5 climate change scenario^59^; data sources include WorldClim^30,31^, ENVIREM^60^, and the ETCCDI extremes indices derived by Sillmann *et al*.^61,62^ from ERA-40^63^ and CCSM4^64^. Additionally, present climate is classified via the Köppen-Geiger climate classification system, from GLOH2O^55^. Geological data include soil types, from the Digital Soil Map of the World (DSMW)^65^; near-surface rock types, from the Global Lithological Map (GLiM)^66^; and sedimentary basin types^67^. Human geography is quantified by human population density, from the Gridded Population of the World (GPW)^68^. Land cover is described by the International Geosphere-Biosphere Programme (IGBP) cover classification scheme, from MCD12Q1^69^; and by percent tree, non-tree, and non-vegetated cover, from MOD44B^70^. The Earth’s physical geography is characterized by continental and island landmasses, from Natural Earth; lakes and wetlands, from the Global Lakes and Wetlands Database (GLWD)^71^; biogeographic realms^56^; and terrestrial topography and ocean bathymetry, from ENVIREM^60^ and SRTM30_PLUS^72^. Finally, distributional data include the present and estimated natural ranges of large mammalian species, from the IUCN Red List^50^ and the Phylogenetic Atlas of Mammal Macroecology (PHYLACINE)^73,74^.

#### Climate

- **ENVIREM**. The ENVIREM (ENVIronmental Rasters for Ecological Modeling) dataset^60^ contains 16 climatic variables derived from WorldClim v1.4 monthly temperature and precipitation^30^, and extraterrestrial radiation. These are intended to compliment the WorldClim v1.4 bioclimatic variables^30^, capturing additional environmental features directly relevant to floral and faunal physiology and ecology^60^. Source rasters at 30 arc-second resolution were summarized by area-weighted mean at ISEA3H resolutions 8 and 9. Variable codes, descriptions, and units are listed in Table 2. Title and Bemmels^60^, and references therein, provide full definitions and calculation methods for these variables.

**Table 2:** Codes, descriptions, and units for the 16 ENVIREM climatic variables, from Title and Bemmels^60^.

Code	Description	Units
AnnualPET	Annual Potential Evapotrans.	mm/Year
AridityIndexThornthwaite	Thornthwaite Aridity Index	—
ClimaticMoistureIndex	Climatic Moisture Index	—
Continentality	Mean Temp. Warmest Coldest	°C
EmbergerQ	Emberger Pluviothermic Quotient	—
GrowingDegDays0	Sum Mean Temp. 0 °C Days	—
GrowingDegDays5	Sum Mean Temp. 5 °C Days	—
MaxTempColdest	Max. Temp. Coldest Month	0.1 °C
MinTempWarmest	Min. Temp. Warmest Month	0.1 °C
MonthCountByTemp10	Months Mean Temp. 10 °C	Months
PETColdestQuarter	Mean PET Coldest Quarter	mm/Month
PETDriestQuarter	Mean PET Driest Quarter	mm/Month
PETSeasonality	PET Seasonality	mm/Month
PETWarmestQuarter	Mean PET Warmest Quarter	mm/Month
PETWettestQuarter	Mean PET Wettest Quarter	mm/Month
ThermicityIndex	Compensated Thermicity Index	°C
- **ETCCDI**. A comprehensive set of 27 climate extremes indices was defined by the Expert Team on Climate Change Detection and Indices (ETCCDI); these generally capture “moderate” extremes, having recurrence intervals of a year or shorter, and are based on observed/simulated daily temperature and precipitation^61,62^. Sillmann *et al*.^61,62^ derive these indices from results of a number of global climate models and atmospheric reanalyses, several of which were incorporated in the Eco-ISEA3H database^37^. Given the relatively low-resolution grids used in modeling and reanalysis, these source rasters were interpolated to ISEA3H cell centroids by inverse (geodesic) distance weighting (IDW). Variable codes, descriptions, and units are listed in Table 3. Sillmann *et al*.^61^ provide full definitions and calculation methods for these indices.

**Table 3:** Codes, descriptions, and units for the 27 ETCCDI climate extremes indices, from Sillmann *et al*.^61,62^.

Code	Description	Units
CDD	Max. Length Dry Spell	Days
CSDI	Cold Spell Duration Index	Days
CWD	Max. Length Wet Spell	Days
DTR	Daily Temp. Range	°C
FD	Frost Days	Days
GSL	Growing Season Length	Days
ID	Icing Days	Days
PRCPTOT	Annual Precip.	mm
R1MM	Annual Days Precip. ≥1MM	Days
R10MM	Annual Days Precip. ≥10MM	Days
R20MM	Annual Days Precip. ≥20MM	Days
R95P	Annual Sum Daily Precip. > 95th	mm
R99P	Annual Sum Daily Precip. > 99th	mm
RX1DAY	Max. 1-Day Precip.	mm
RX5DAY	Max. Consec. 5-Day Precip.	mm
SDII	Simple Precip. Intensity Index	mm/Day
SU	Summer Days	Days
TN10P	% Days TN < 10th Percentile	Percent
TN90P	% Days TN > 90th Percentile	Percent
TNN	Min. Daily Min. Temp.	°C
TNX	Max. Daily Min. Temp.	°C
TR	Tropical Nights	Days
TX10P	% Days TX < 10th Percentile	Percent
TX90P	% Days TX > 90th Percentile	Percent
TXN	Min. Daily Max. Temp.	°C
TXX	Max. Daily Max. Temp.	°C
WSDI	Warm Spell Duration Index	Days

The Eco-ISEA3H database^37^ includes ETCCDI variables based on results of the ERA-40 reanalysis^63^, produced by the European Centre for Medium-Range Weather Forecasts (ECMWF). The reanalysis combines past meteorological observations with a weather forecasting model, producing a global representation of the state of the atmosphere for each reanalysis time step, usually a six-hour interval^63^. These were averaged for the period 1958 to 2001, the 44 full years for which the ERA-40 reanalysis was conducted, and were interpolated to ISEA3H resolutions 5 to 9.

Additionally, the database includes ETCCDI variables based on results of the Community Climate System Model v4 (CCSM4), a global climate model developed for CMIP5^64^. These were averaged for the period 1950 to 2000, to match the approximate period covered by WorldClim v1.4, and for the period 2061 to 2080, to match the final interval for which CCSM4 model results were downscaled/debiased using WorldClim v1.4^30^. Variables were interpolated to ISEA3H resolution 9.

ETCCDI variables for this latter period represent conditions under Representative Concentration Pathway (RCP) 8.5, the RCP resulting in the highest radiative forcing (8.5 W/m^2^) by 2100^59^. This scenario was selected such that future conditions maximally different from the present might be considered; in RCP 8.5, rapid population growth, and relatively slow growth in per capita income and technological development, lead to high energy demand without associated climate mitigation policies, resulting in greenhouse gas emissions and atmospheric concentrations increasing significantly in the coming decades^59^.

- **Köppen-Geiger Climate Classification**. As implemented by Beck *et al*.^55^, the Köppen-Geiger system classifies the Earth’s terrestrial climates into five primary classes, and further into 30 subclasses, based on a set of threshold criteria referencing monthly mean temperature and precipitation. These climate classes are ecologically significant, as regions within each class support floral communities sharing common characteristics. Beck *et al*.^55^ utilize four climatic datasets, including WorldClim v1.x and v2.x, adjusted to the period 1980 to 2016, to define the present-day classes incorporated in the Eco-ISEA3H database^37^. The source raster at 30 arc-second resolution was summarized by fraction and mode at ISEA3H resolution 9. Variable codes and descriptions are listed in Table 4.

**Table 4:** Codes and descriptions for the 30 Köppen-Geiger climate classes, from Beck *et al*.^55^.

Code	Class
Af	Tropical, Rainforest
Am	Tropical, Monsoon
Aw	Tropical, Savannah
BWh	Arid, Desert, Hot
BWk	Arid, Desert, Cold
BSh	Arid, Steppe, Hot
BSk	Arid, Steppe, Cold
Csa	Temperate, Dry Summer, Hot Summer
Csb	Temperate, Dry Summer, Warm Summer
Csc	Temperate, Dry Summer, Cold Summer
Cwa	Temperate, Dry Winter, Hot Summer
Cwb	Temperate, Dry Winter, Warm Summer
Cwc	Temperate, Dry Winter, Cold Summer
Cfa	Temperate, w/o Dry Season, Hot Summer
Cfb	Temperate, w/o Dry Season, Warm Summer
Cfc	Temperate, w/o Dry Season, Cold Summer
Dsa	Cold, Dry Summer, Hot Summer
Dsb	Cold, Dry Summer, Warm Summer
Dsc	Cold, Dry Summer, Cold Summer
Dsd	Cold, Dry Summer, Very Cold Winter
Dwa	Cold, Dry Winter, Hot Summer
Dwb	Cold, Dry Winter, Warm Summer
Dwc	Cold, Dry Winter, Cold Summer
Dwd	Cold, Dry Winter, Very Cold Winter
Dfa	Cold, w/o Dry Season, Hot Summer
Dfb	Cold, w/o Dry Season, Warm Summer
Dfc	Cold, w/o Dry Season, Cold Summer
Dfd	Cold, w/o Dry Season, Very Cold Winter
ET	Polar, Tundra
EF	Polar, Frost
- **WorldClim v1.4**. The first-generation WorldClim dataset^30^ contains four monthly themes, each with 12 variables, characterizing monthly temperature and precipitation; additionally, it contains 19 bioclimatic variables, derived from the monthly variables, capturing biologically relevant seasonal and annual features of the climate system. These bioclimatic variables, first developed for the BIOCLIM species distribution modeling (SDM) package^75^, are used extensively in SDM studies; a recent synthesis found most were included in more than 1,000 published MaxEnt SDMs (of 2,040 reviewed)^76^.

WorldClim monthly temperature and precipitation rasters are interpolated from weather station observations averaged for the approximate period 1950 to 2000. The interpolation was done using thin plate smoothing splines, with latitude, longitude, and elevation as predictor variables^30^. These rasters characterize present-day climate, and further served as an observational baseline with which the predictions of CMIP5 global climate models were downscaled and bias-corrected.

The 19 bioclimatic variables, for both present-day and future conditions (the latter averaged for the period 2061 to 2080, from the CCSM4 RCP 8.5 simulation), were incorporated into the Eco-ISEA3H database^37^; source rasters at 30 arc-second resolution were summarized by area-weighted mean at ISEA3H resolution 9. Variable codes, descriptions, and units are listed in Table 5. O’Donnell and Ignizio^77^ provide full definitions and calculation methods for these variables.

**Table 5:** Codes, descriptions, and units for the 19 WorldClim bioclimatic variables, from v1.4^30^ and v2.0^31^.

Code	Description	Units (v1.4)	Units (v2.0)
BIO01	Annual Mean Temp.	0.1 °C	°C
BIO02	Mean Diurnal Range	0.1 °C	°C
BIO03	Isothermality	Percent	Percent
BIO04	Temp. Seasonality	0.001 °C	0.01 °C
BIO05	Max. Temp. Warmest Month	0.1 °C	°C
BIO06	Min. Temp. Coldest Month	0.1 °C	°C
BIO07	Annual Temp. Range	0.1 °C	°C
BIO08	Mean Temp. Wettest Quarter	0.1 °C	°C
BIO09	Mean Temp. Driest Quarter	0.1 °C	°C
BIO10	Mean Temp. Warmest Quarter	0.1 °C	°C
BIO11	Mean Temp. Coldest Quarter	0.1 °C	°C
BIO12	Annual Precip.	mm	mm
BIO13	Precip. Wettest Month	mm	mm
BIO14	Precip. Driest Month	mm	mm
BIO15	Precip. Seasonality	Percent	Percent
BIO16	Precip. Wettest Quarter	mm	mm
BIO17	Precip. Driest Quarter	mm	mm
BIO18	Precip. Warmest Quarter	mm	mm
BIO19	Precip. Coldest Quarter	mm	mm

- **WorldClim v2.0**. The second-generation WorldClim dataset^31^ contains seven monthly themes, each with 12 variables, characterizing monthly temperature, precipitation, solar radiation, wind speed, and vapor pressure; additionally, it contains the standard set of 19 bioclimatic variables, derived from monthly temperature and precipitation.

As in the first-generation dataset, monthly rasters were interpolated from weather station observations, averaged here for the approximate period 1970 to 2000^31^. Again, thin plate smoothing splines were used in the interpolation, but with additional covariates included for one or more interpolated features: distance to coast, computed extraterrestrial radiation, and three satellite-derived observations - cloud cover, and maximum and minimum land surface temperature, from the Moderate Resolution Imaging Spectroradiometer (MODIS) instrument.

The 12 source rasters for each of the seven monthly themes, at 30 arc-second resolution, were summarized by centroid at ISEA3H resolutions 5 to 10. Additionally, the 19 source bioclimatic rasters, at 30 arc-second resolution, were summarized by centroid at ISEA3H resolutions 5 to 10, and by area-weighted mean at ISEA3H resolutions 6 to 9. Codes, descriptions, and units for the bioclimatic variables are listed in Table 5.

#### Geol10ogy

- **DSMW**. The Digital Soil Map of the World (DSMW)^65^ describes the geographic distribution and physical and chemical properties of the world’s soils. The DSMW was digitized from the FAO-UNESCO Soil Map of the World, printed at 1:5,000,000 scale. Each digitized mapping unit is assigned a number of soil attributes; here we classify units via the DOMSOI attribute, the dominant soil or land unit code. The DSMW includes 117 soils in 26 major soil groupings, as well as six other land units, for a total of 123 DOMSOI classes. The source vector dataset was summarized by fraction and mode at ISEA3H resolutions 5, 6, and 9. Variable codes and descriptions are listed in Table 6.

**Table 6:** Codes and descriptions for the 123 DSMW soil and land units, from the FAO^65^.

Code	Class	Code	Class	Code	Class
Af	Ferric Acrisols	Hh	Haplic Phaeozems	RK	Rock Debris or Desert Detritus
Ag	Gleyic Acrisols	Hl	Luvic Phaeozems	Rc	Calcaric Regosols
Ah	Humic Acrisols	I	Lithosols	Rd	Dystric Regosols
Ao	Orthic Acrisols	J	Fluvisols	Re	Eutric Regosols
Ap	Plinthic Acrisols	Jc	Calcaric Fluvisols	Rx	Gelic Regosols
Bc	Chromic Cambisols	Jd	Dystric Fluvisols	S	Solonetz
Bd	Dystric Cambisols	Je	Eutric Fluvisols	ST	Salt Flats
Be	Eutric Cambisols	Jt	Thionic Fluvisols	Sg	Gleyic Solonetz
Bf	Ferralic Cambisols	K	Kastanozems	Sm	Mollic Solonetz
Bg	Gleyic Cambisols	Kh	Haplic Kastanozems	So	Orthic Solonetz
Bh	Humic Cambisols	Kk	Calcic Kastanozems	Th	Humic Andosols
Bk	Calcic Cambisols	Kl	Luvic Kastanozems	Tm	Mollic Andosols
Bv	Vertic Cambisols	L	Luvisols	To	Ochric Andosols
Bx	Gelic Cambisols	La	Albic Luvisols	Tv	Vitric Andosols
C	Chernozems	Lc	Chromic Luvisols	U	Rankers
Cg	Glossic Chernozems	Lf	Ferric Luvisols	V	Vertisols
Ch	Haplic Chernozems	Lg	Gleyic Luvisols	Vc	Chromic Vertisols
Ck	Calcic Chernozems	Lk	Calcic Luvisols	Vp	Pellic Vertisols
Cl	Luvic Chernozems	Lo	Orthic Luvisols	W	Planosols
DS	Dunes or Shifting Sands	Lp	Plinthic Luvisols	WR	Inland Water or Ocean
Dd	Dystric Podzoluvisols	Lv	Vertic Luvisols	Wd	Dystric Planosols
De	Eutric Podzoluvisols	Mg	Gleyic Greyzems	We	Eutric Planosols
Dg	Gleyic Podzoluvisols	Mo	Orthic Greyzems	Wh	Humic Planosols
E	Rendzinas	ND	No Data	Wm	Mollic Planosols
Fa	Acric Ferralsols	Nd	Dystric Nitosols	Ws	Solodic Planosols
Fh	Humic Ferralsols	Ne	Eutric Nitosols	X	Xerosols
Fo	Orthic Ferralsols	Nh	Humic Nitosols	Xh	Haplic Xerosols
Fp	Plinthic Ferralsols	O	Histosols	Xk	Calcic Xerosols
Fr	Rhodic Ferralsols	Od	Dystric Histosols	Xl	Luvic Xerosols
Fx	Xanthic Ferralsols	Oe	Eutric Histosols	Xy	Gypsic Xerosols
G	Gleysols	Ox	Gelic Histosols	Y	Yermosols
GL	Glacier	Pg	Gleyic Podzols	Yh	Haplic Yermosols
Gc	Calcaric Gleysols	Ph	Humic Podzols	Yk	Calcic Yermosols
Gd	Dystric Gleysols	Pl	Leptic Podzols	Yl	Luvic Yermosols
Ge	Eutric Gleysols	Po	Orthic Podzols	Yt	Takyric Yermosols
Gh	Humic Gleysols	Pp	Placic Podzols	Yy	Gypsic Yermosols
Gm	Mollic Gleysols	Qa	Albic Arenosols	Z	Solonchaks
Gp	Plinthic Gleysols	Qc	Cambic Arenosols	Zg	Gleyic Solonchaks
Gx	Gelic Gleysols	Qf	Ferralic Arenosols	Zm	Mollic Solonchaks
Hc	Calcaric Phaeozems	Ql	Luvic Arenosols	Zo	Orthic Solonchaks
Hg	Gleyic Phaeozems	R	Regosols	Zt	Takyric Solonchaks
- **GLiM**. The Global Lithological Map (GLiM)^66^ represents the rock and unconsolidated sediments at or near the Earth’s terrestrial surface; this geological material is a source of geochemical flux to the Earth’s soils, biosphere, and hydrosphere. Hartmann and Moosdorf^66^ compiled the map and accompanying database from 92 regional geological maps and 318 literature sources. Rock was classified into 16 first-level lithological classes; 12 second-level and 14 third-level subclasses further describe specific mineralogical and physical properties.

The source vector dataset was summarized by centroid at ISEA3H resolution 9. Variable codes and descriptions are listed in Table 7. The attribute assigned each ISEA3H cell takes the form *xxyyzz*; underscore characters (_) in the *yy* and/or *zz* slots indicate the second- and/or third-level subclasses were undefined.

**Table 7:** Codes and descriptions for the 16 first-level, 12 second-level, and 14 third-level GLiM lithological classes, from Hartmann and Moosdorf^66^.

Code	Class
**1st Level (xx)**	
ev	Evaporites
ig	Ice and Glaciers
mt	Metamorphics
nd	No Data
pa	Acid Plutonic Rocks
pb	Basic Plutonic Rocks
pi	Intermediate Plutonic Rocks
py	Pyroclastics
sc	Carbonate Sedimentary Rocks
sm	Mixed Sedimentary Rocks
ss	Siliciclastic Sedimentary Rocks
su	Unconsolidated Sediments
va	Acid Volcanic Rocks
vb	Basic Volcanic Rocks
vi	Intermediate Volcanic Rocks
wb	Water Bodies
**2nd Level (yy)**	
ad	Alluvial Deposits
am	Mafic Metamorphics Mentioned
ds	Dune Sands
gr	Greenstone Mentioned
la	Laterites
lo	Loess
mx	Mixed Grain Size
or	Organic Sediment
pu	(Pure) Carbonate
py	Pyroclastics Mentioned
sh	Fine Grained
ss	Coarse Grained
**3rd Level (zz)**	
bs	Black Shale Mentioned
ch	Chert Mentioned
cl	Fossil Plant Organic Material Mentioned
ev	Subordinate Evaporites Mentioned
fe	Reduced-Iron Minerals Mentioned
gl	Glacial Influence Mentioned
mt	Metamorphic Influence Mentioned
ph	Phosphorous-Rich Minerals Mentioned
pr	Subordinate Plutonics Mentioned
pt	Pyrite Mentioned
sr	Subordinate Sedimentary Rocks Mentioned
su	Subordinate Unconsolidated Sediments Mentioned
vr	Subordinate Volcanics Mentioned
we	Intensive Weathering

- **Sedimentary Basins**. Sedimentary basins are areas of subsidence in the Earth’s crust, in which sediments eroded from uplands are deposited and potentially preserved for a million or more years^67^, thus entering the planet’s long-term geological record. Nyberg and Howell^67^ delineate active sedimentary basins, covering both the Earth’s terrestrial surface and marine areas over continental crust. The authors operationally defined basins as low-relief areas containing Quaternary Period sediments, and further classified the basins by tectonic setting, identifying backarc, forearc, foreland, extensional, intracratonic, passive margin, and strike-slip basins on the basis of published literature and geological maps^67^.

Terrestrial basins were incorporated in the Eco-ISEA3H database^37^. Note that no terrestrial backarc basins were delineated. The source vector dataset was summarized by fraction and mode at ISEA3H resolution 9.

#### Human geography

- **GPW**. Human population density is one of several measures of human presence and activity which together define the human “footprint,” associated with profound, adverse effects on natural systems^78^. Given this pervasive impact, data characterizing degree of human influence are used as predictors in some ecological models, including SDMs^28^. The Gridded Population of the World (GPW)^68^ density dataset represents the global distribution of human population density, developed using census records, population registers, and the administrative boundaries of approximately 13.5 million national and subnational units. Density, measured by population count per square kilometer, was estimated every five years, from 2000 to 2020, inclusive. The source raster dataset for each year, at 30 arc-second resolution, was summarized by area-weighted mean at ISEA3H resolutions 6 to 9.

#### Land cover

- **MCD12Q1**. The Moderate Resolution Imaging Spectroradiometer (MODIS) land cover type (MCD12Q1) dataset^69^ describes land cover globally, via six different classification schemes. The Eco-ISEA3H database^37^ includes land cover classified via the International Geosphere-Biosphere Programme (IGBP) scheme, initially developed for the DISCover dataset^79^; the IGBP scheme includes 16 land cover classes, 13 natural and three anthropogenically modified. The MCD12Q1 dataset is derived from reflectance data collected by the MODIS instruments aboard the Terra and Aqua satellites; the two instruments observe the entirety of the Earth’s surface every one to two days, recording reflectance in 36 spectral bands.

MCD12Q1 land cover is estimated annually. For each year, reflectance time-series data are smoothed and gap-filled via smoothing splines; derived spectro-temporal features are used as input to a random forest classifier; and output land cover classifications are post-processed, to incorporate prior knowledge and reduce inter-annual variability^69^. The source raster dataset for 2001 and 2014 to 2018, inclusive, at approximately 500 meter resolution, was summarized by centroid, fraction, and mode at ISEA3H resolutions 5 to 10. Variable codes and descriptions are listed in Table 8.

**Table 8:** Codes and descriptions for the 16 IGBP land cover classes, from Friedl and Sulla-Menashe^69^.

Code	Class
01	Evergreen Needleleaf Forests
02	Evergreen Broadleaf Forests
03	Deciduous Needleleaf Forests
04	Deciduous Broadleaf Forests
05	Mixed Forests
06	Closed Shrublands
07	Open Shrublands
08	Woody Savannas
09	Savannas
10	Grasslands
11	Permanent Wetlands
12	Croplands
13	Urban and Built-up Lands
14	Cropland/Natural Vegetation Mosaics
15	Permanent Snow and Ice
16	Barren

**MOD44B**. The MODIS vegetation continuous fields (VCF) dataset (MOD44B)^70^ describes global land cover quantitatively, as fractions of three cover components: tree canopy, non-tree canopy, and non-vegetated, barren cover. Note that *canopy* cover, as defined here, indicates the area over which light is intercepted; this differs from *crown* cover, which indicates the area covered by a plant’s crown regardless of light interception/penetration. The MOD44B dataset is derived from reflectance data collected by the MODIS instrument aboard the Terra satellite; for each annual VCF estimate, reflectance time-series data are used as input to a bagged ensemble of linear regression trees^70^. The source raster dataset for 2018, at approximately 250 meter resolution, was summarized by area-weighted mean at ISEA3H resolution 9.

#### Physical geography

- **Biogeographic Realms**. As defined by Olson *et al*.^56^, the eight terrestrial biogeographic realms are the broadest divisions of the Earth’s terrestrial flora and fauna; these may be further subdivided into biomes and ecoregions, the latter containing distinct natural communities. Olson *et al*.^56^ developed this hierarchical system primarily for global and regional conservation planning. Realm, biome, and ecoregion delineations are based on expert knowledge, contributed by more than 1,000 scientists working in relevant fields; these divisions thus incorporate knowledge of endemic taxa, unique species assemblages, and local geological and biogeographical history^56^. Realms were included in the Eco-ISEA3H database^37^ to provide a high-level classification of the Earth’s biogeography, from a source frequently cited in the scientific literature. The source vector dataset was summarized by fraction and mode at ISEA3H resolutions 5 to 9. Variable codes and descriptions are listed in Table 9.

**Table 9:** Codes and descriptions for the eight biogeographic realms, from Olson *et al*.^56^.

Code	Class
AA	Australasia
AN	Antarctic
AT	Afrotropic
IM	Indo-Malay
NA	Nearctic
NT	Neotropic
OC	Oceania
PA	Palearctic
- **ENVIREM**. In addition to the climatic variables discussed previously, the ENVIREM dataset^60^ contains two topographic variables, derived from SRTM30_PLUS. These two indices characterize *terrain roughness*, a measure of variability in local elevation; and *topographic wetness*, a function of slope and upgradient contributing area. Source rasters at 30 arc-second resolution were summarized by area-weighted mean at ISEA3H resolutions 8 and 9. Variable codes, descriptions, and units are listed in Table 10.

**Table 10:** Codes, descriptions, and units for the two ENVIREM topographic variables, from Title and Bemmels^60^.

Code	Description	Units
TRI	Terrain Roughness Index	—
TopoWet	Topographic Wetness Index	—
- **GLWD**. The Global Lakes and Wetlands Database (GLWD)^71^, Level 3, represents the maximum extent of lakes, reservoirs, rivers, and a number of wetland types, comprising 12 waterbody classes in total. Lehner and Döll^71^ compiled the three levels of the GLWD by combining seven source map and attribute datasets, and suggest Level 3 may be useful as input in global hydrologic and climatic modeling. The source raster dataset at 30 arc-second resolution was summarized by fraction and mode at ISEA3H resolution 9. Variable codes and descriptions are listed in Table 11.

**Table 11:** Codes and descriptions for the 12 GLWD waterbody classes, from Lehner and Döll^71^.

Code	Class
01	Lake
02	Reservoir
03	River
04	Freshwater Marsh, Floodplain
05	Swamp Forest, Flooded Forest
06	Coastal Wetland
07	Pan, Brackish/Saline Wetland
08	Bog, Fen, Mire
09	Intermittent Wetland/Lake
10	50 - 100% Wetland
11	25 - 50% Wetland
12	Wetland Complex (0 - 25%)
- **Natural Earth**. Natural Earth is a public-domain collection of raster and vector datasets developed for production cartography. Three vector themes describing physical geography were incorporated: *Land*, which includes continents and major islands; *Islands*, which includes additional minor islands; and *Lakes*, which includes lakes and reservoirs. Source vector datasets at 1:10,000,000 scale were summarized by fraction at ISEA3H resolutions 5 to 9. Further, fractions for a *Terra* theme were calculated, by adding per-cell *Land* and *Islands*, and subtracting *Lakes*. The *Terra* theme may be thresholded (for example, at a fraction value ≥0.5) to identify terrestrial ISEA3H cells, excluding cells covered primarily by ocean or freshwater habitat.
- **SRTM30_PLUS**. The SRTM30_PLUS dataset^72^ is a global digital elevation model (DEM), representing the Earth’s terrestrial topography and ocean bathymetry. A number of elevation sources were incorporated in developing the DEM; terrestrial topography was derived from the Shuttle Radar Topography Mission (SRTM) at latitudes between ±60°, from GTOPO30 in the Arctic, and from GLAS/ICESat in the Antarctic. Ocean bathymetry was derived from satellite radar altimetry, calibrated on 298 million corrected ship-based depth soundings, gathered from several sounding sources^72^. The source raster dataset at 30 arc-second resolution was summarized by area-weighted mean at ISEA3H resolutions 6 to 10.

#### Species ranges

From the Red List and the Phylogenetic Atlas, the geographic ranges of species belonging to four mammalian orders were sampled: Artiodactyla (even-toed ungulates), Perissodactyla (odd-toed ungulates), Primates, and Proboscidea (elephants). These species are primarily large-bodied herbivores, and as such are frequently the subject of dental ecometrics research; for example, averaged dental traits of communities of these mammals have been used to predict measures of local precipitation, at both global^3^ and regional^11^ scales.

- **IUCN Red List**. The International Union for Conservation of Nature’s (IUCN) Red List of Threatened Species^50^ comprises global assessments of the conservation status of nearly 150,000 floral, faunal, and fungal species. The Red List includes expert-delineated geographic ranges for most of these species, including most extant mammalian species. For each species, portions of the range for which the species’ presence was coded *extant*, and for which its origin was coded *native* or *reintroduced*, were sampled. Source vector datasets were summarized by fraction at ISEA3H resolutions 8 to 9 (Artiodactyla and Perissodactyla), 9 (Primates), and 7 to 9 (Proboscidea).
- **PHYLACINE**. The Phylogenetic Atlas of Mammal Macroecology (PHYLACINE)^73,74^ includes trait, phylogeny, and geographic range data for all mammalian species known from the last interglacial period (approximately 130,000 years ago) to the present, both extant and recently extinct. PHYLACINE includes species’ ranges under two scenarios, both of which were incorporated: present-day ranges, from the IUCN v2016.3; and “present-natural” ranges, for which each species’ present-day range was modified to estimate its distribution under current climatic conditions, but absent anthropogenic pressure. This included, among eight modification categories, reconnecting fragmented ranges, by filling suitable intervening habitat; and expanding ranges reduced by human activity, by filling suitable adjacent habitat. Present-natural range modifications are documented for each species in PHYLACINE’s metadata, and intended to mitigate human impact on the results of macroecological analysis and modeling. Source rasters at approximately 100 kilometer resolution were summarized by centroid at ISEA3H resolution 9.

## Data Records

The Eco-ISEA3H database^37^ and accompanying metadata are available at Fairdata.fi, a digital preservation service of the Finnish Ministry of Education and Culture, produced by the Finnish IT Center for Science (CSC). The database may be accessed via the following DOI: 10.23729/37d3e51e-3bf0-453a-a2ab-ed1a935ccaf8.

### Eco-ISEA3H themes & variables

The Eco-ISEA3H database^37^ contains 3,033 variables, derived from source dataset themes and component classes and/or variables characterizing climate, geology, land cover, physical and human geography, and the geographic ranges of large mammalian species. Eco-ISEA3H themes and variables are summarized in Table 12.

**Table 12:** Summary statistics compiled for the Eco-ISEA3H database^37^. Each is described by the source dataset name, version, and scenario from which it was derived; the temporal period it represents, as well as the temporal resolution of the source dataset and derived statistic; the source theme from which it was derived; the statistic(s) and ISEA3H resolution(s) by which the source theme, classes, and/or variables were summarized; and finally the null value used to indicate missing data.

Dataset	Version	Scenario	Period	Source Resolution	Eco-ISEA3H Resolution	Theme	Summary Statistic(s)	ISEA Resolution(s)	Null Value
**Climate**									
Community Climate System Model Version 4 (CCSM4)	.	Historical	1950–2000	Annual	Single Summary	Expert Team on Climate Change Detection & Indices (ETCCDI) Climate Extremes Indices	IDW	9	.
	.	RCP 8.5	2061–2080	Single Summary	Single Summary	Bioclimatic Variables (BIO)	Mean	9	−1000
European Centre for Medium-Range Weather Forecasts (ECMWF)	ERA-40	.	1958–2001	Annual	Single Summary	Expert Team on Climate Change Detection & Indices (ETCCDI) Climate Extremes Indices	IDW	5–9	.
ENVIronmental Rasters for Ecological Modeling (ENVIREM)	1.0	.	1950–2000	Single Summary	Single Summary	Climatic Variables	Mean	8–9	−1000
GLOH2O	1.0	.	1980–2016	Single Summary	Single Summary	Köppen-Geiger Climate Classification	Fraction, Mode	9	NA
WorldClim	1.4	.	1950–2000	Single Summary	Single Summary	Bioclimatic Variables (BIO)	Mean	9	−1000
	2.0	.	1970–2000	Single Summary	Single Summary	Bioclimatic Variables (BIO)	Mean	6–9	−100
						Bioclimatic Variables (BIO)	Centroid	5–10	−100
						Monthly Precipitation (PREC)			−1
						Monthly Solar Radiation (SRAD)			−1
						Monthly Mean Temperature (TAVG)			−100
						Monthly Minimum Temperature (TMIN)			−100
						Monthly Maximum Temperature (TMAX)			−100
						Monthly Vapor Pressure (VAPR)			−1
						Monthly Wind Speed (WIND)			−1
**Geology**									
Digital Soil Map of the World (DSMW)	3.6	.	.	.	.	Soil Units	Fraction, Mode	5–6, 9	−1
Global Lithological Map (GLiM)	1.1	.	.	.	.	Lithology	Centroid	9	—
Sedimentary Basins	.	.	.	.	.	Structure	Fraction	9	.
**Human Geography**									
Gridded Population of the World (GPW)	4.11	.	2000, 2005, 2010, 2015, 2020	Annual	Annual	Population Density	Mean	6–9	−1
**Land Cover**									
MCD12Q1	6.0	.	2001, 2014–2018	Annual	Annual	International Geosphere-Biosphere Programme (IGBP) Land Cover Classification	Centroid, Fraction, Mode	5–10	−1
MOD44B	6.0	.	2018	Annual	Annual	Vegetation Continuous Fields (VCF)	Mean	9	−1
**Physical Geography**									
ENVIronmental Rasters for Ecological Modeling (ENVIREM)	1.0	.	.	.	.	Topographic Variables	Mean	8–9	−1000
Natural Earth (NE)	4.1.0	.	.	.	.	Lakes	Fraction	5–9	.
						Land			
						Minor Islands			
						Terra			
SRTM30-PLUS	11	.	.	.	.	Elevation	Mean	6–10	.
World Wildlife Fund (WWF) Terrestrial Ecoregions (TE)	2.0	.	.	.	.	Biogeographic Realms	Fraction, Mode	5–9	−1
World Wildlife Fund (WWF) Global Lakes & Wetlands Database (GLWD)	1.0	.	.	.	.	Level 3	Fraction, Mode	9	−1
**Species Ranges**									
Red List (RL) of Threatened Species	2019.1	.	.	.	.	Artiodactyla: Antilocapridae, Bovidae, Camelidae, Cervidae, Giraffidae, Hippopotamidae, Moschidae, Suidae, Tayassuidae, Tragulidae	Fraction	8–9	.
						Perissodactyla: Equidae, Rhinocerotidae, Tapiridae	Fraction	8–9	.
						Primates: Aotidae, Atelidae, Callitrichidae, Cebidae, Cercopithecidae, Cheirogaleidae, Daubentoniidae, Galagidae, Hominidae, Hylobatidae, Indriidae, Lemuridae, Lepilemuridae, Lorisidae, Pitheciidae, Tarsiidae	Fraction	9	.
						Proboscidea: Elephantidae	Fraction	7–9	.
Phylogenetic Atlas of Mammal Macroecology (PHYLACINE)	1.2.1	Present	.	.	.	Artiodactyla	Centroid	9	.
						Perissodactyla			
						Primates			
						Proboscidea			
		Present Natural	.	.	.	Artiodactyla	Centroid	9	.
						Perissodactyla			
						Primates			
						Proboscidea			

Note that while several source datasets represent the present-day generally, others represent a specific temporal period, and have a set temporal resolution. Such datasets, and Eco-ISEA3H variables derived from these datasets, fall into three categories. Certain source climatic datasets represent (1) single-value summaries over a multi-year period, and were incorporated as such: ENVIREM climate variables^60^, Köppen-Geiger climate classes^55^, and WorldClim v1.4^30^ and v2.0^31^ variables (both historical interpolations and downscaled CCSM4 projections). Certain other source datasets represent (2) a time-series of annual observations, and were incorporated as such: GPW human population density^68^, MCD12Q1 land cover classes^69^, and MOD44B vegetation variables^70^. Finally, certain source climatic datasets again represent (3) a time-series of annual observations, but were incorporated as multi-year summaries: ETCCDI extremes indices^61,62^ from CCSM4^64^ and ECMWF^63^. The periods over which CCSM4 annual results were summarized were selected to match the multi-year summary periods of WorldClim v1.4.

Further note that several source datasets contain no-data regions, primarily over the world’s oceans; WorldClim v1.4^30^ and v2.0^31^, for example, are clipped to the Earth’s terrestrial surface. Summary statistics in ISEA3H cells over these regions are similarly null, and must be assigned a value indicating missing data, outside the range of values taken by the theme or variable. Where necessary, these null values are listed in Table 12. The proportion of data values to null values in these datasets is less than or equal to the proportion of land to ocean, approximately 3:7.

### Directory structure & file naming convention

To facilitate use by a wide range of researchers in the biological, geological, and computational sciences, development of the Eco-ISEA3H database^37^ was guided by FAIR principles for scientific data stewardship^80^. To maximize interoperability, the database comprises plain-text, tab-delimited files, organized within a regular directory structure. The names of folders, files, and column headers within files follow a standard format, each a concatenation of regular components separated by underscores.

At the root of the Eco-ISEA3H directory structure, each ISEA3H resolution has an associated folder. Within these is a folder for each source dataset sampled at that resolution, named following the format:


[Source Dataset]_V[Version Number]


Within each dataset folder are one or more text files, each containing data related to one of the dataset’s scientific *themes*. Discrete themes comprise one or more *classes*, continuous themes one or more real-valued *variables*. Text files, each containing a per-cell statistical summary of a theme or its components, are named following the format:


ISEA3H[Resolution]_[Source Dataset]_V[Version Number]_Y[Year]_[Theme]_[Summary Statistic]


Filenames may include zero, one, or two consecutive Y[*Year*] components, based on the temporal scope of the source dataset. For datasets without a defined temporal period (for example, the Global Lithological Map^66^), or averaged over a single, standard period (for example, WorldClim v2.0^31^), no Y[*Year*] components are required. A single Y[*Year*] component indicates the single year represented by the source dataset, two components the temporal range, inclusive, represented.

The hexagon ID, or HID, is included in all text files, and serves as primary key for the Eco-ISEA3H database^37^. HIDs uniquely identify each hexagonal cell within each ISEA3H resolution, and may be used to link records associated with each cell among the database’s spatial and tabular files; see the vignette for an example of linking via the *merge* function in R.

#### Discrete themes

Summary values of discrete, categorical themes are named following the format:


[Theme or Class]_[Summary Statistic]


Alternatively, for themes containing classes identified only by a sequential, integer indexing number, the theme may be added as a prefix, to assemble more informative column headers:


[Theme]_[Class]_[Summary Statistic]


For example, GLOH2O contains a number of scientific themes, one of which, the Köppen-Geiger climate classification^55^, was included in the Eco-ISEA3H database^37^. The theme contains 30 discrete climate classes, each of which was summarized by the *fraction* attribute. Further, the theme as a whole was summarized by the *mode* attribute. Thus, the column containing the first of 30 *fraction* values, indicating the proportion of each ISEA3H cell’s area covered by the *tropical rainforest* climate class (referenced by the code *Af*), was headed *Af_Fraction*. The column containing the theme’s *mode* value, indicating the class covering the greatest proportion of each cell’s area, was headed *KoppenGeiger_Mode*.

#### Continuous variables

Summary values of the component variables of continuous, real-numbered themes are named following the format:


[Variable]_[Summary Statistic]


For example, WorldClim v2.0^31^ contains eight scientific themes, all of which were included in the Eco-ISEA3H database^37^. Monthly precipitation (referenced by the code *PREC*) is one of these. The theme contains a variable for each month, named by appending the month numbers 01 to 12 to the theme’s code. Each of these 12 variables was summarized by the *centroid* attribute. Thus, the variable containing January precipitation was named *PREC01*, and the column containing the *PREC01* variable’s *centroid* value, indicating January precipitation at each ISEA3H cell’s centroid, was headed *PREC01_Centroid*.

## Technical Validation

To validate the operability of the Eco-ISEA3H database^37^, we present a case study in which we assess the bioclimatic envelopes of ten large, widely distributed mammalian species. In ecometric and SDM studies, species’ environmental niches are frequently defined by extracting the values of raster-based environmental variables at locations each species is known or estimated to occur. We assess the degree to which species’ niches may be misrepresented if sampling locations are not equivalent and directly comparable. We contrast our DGGS-based approach, in which environmental conditions are sampled via equal-area hexagonal grid cells, with a baseline approach, in which conditions are sampled via raster pixels of differing geodesic areas. Results of the study highlight differences in perceived niches as measured by the two methods, and support the use of equal-area cells like those of the ISEA3H DGGS.

### Case study: bioclimatic niches

Intuitively, a species’ *niche* describes its place in the environment, the conditions under which it thrives. As operationalized in quantitative ecology, the environment is frequently abstracted, represented by a multi-dimensional environmental space, the axes of which are defined by independent, functionally relevant, often scenopoetic environmental variables^81^. A species’ niche is then the region of this multi-dimensional space (the *n*-dimensional hypervolume^82^) in which the species’ intrinsic rate of population growth is positive^81^. SDMs utilize this niche concept; these models predict species’ occurrence or abundance on the basis of such variables (often climatic and/or topographic^76^), using statistical or ML methods to estimate the species’ response in *n*-dimensional environmental space^28^.

The environmental variables used for SDM training and prediction often derive from raster datasets, commonly developed and distributed in *non-authalic* coordinate reference systems. In such systems, raster pixels vary in area when inversely projected to the Earth’s ellipsoidal surface. For example, WorldClim raster datasets are distributed in the WGS84 coordinate reference system, at 30 arc-second resolution; while raster pixels uniformly measure 30 × 30 arc-seconds, the pixels vary in geodesic area with latitude. Thus, these pixels are not equivalent, directly comparable units of observation.

If non-authalic pixel counts are used for niche analysis or modeling - for example, to determine a species’ probability of occurrence in environmental space, or to quantify changes in a species’ predicted geographic range - results may be considerably biased. However, this is often ignored^83^. To address this problem, the Eco-ISEA3H database^37^ utilizes an equal-area DGGS; at each resolution, the Earth’s surface is partitioned into a set of equal-area hexagonal cells. Here we compare measures of central tendency in species’ niches, as measured by (1) authalic Eco-ISEA3H cells, and (2) a baseline approach, based on the non-authalic pixels of raster datasets in the WGS84 coordinate reference system. We find substantial differences in median bioclimatic values for some large mammalian species, demonstrating the importance of using equal observational units in analysis and modeling.

#### Methods & materials

For this case study, we selected large mammalian species for which the difference between these two approaches was expected to be most extreme. Bias was expected to be greatest where raster pixels having the greatest range of geodesic areas were summarized as if they were equivalent observational units. Budic *et al*.^84^, in a similar SDM study, selected species with northernmost geographic distributions, as projection-induced area distortion is greatest at high latitudes. However, we note that area distortion varies only with latitude; if a species’ range falls within a narrow range of latitude, all raster pixels within the range are distorted similarly, and the pixels are again equivalent. Instead, we sought species with geographic distributions covering the greatest range of latitude, and with distribution centroids most distant from the Equator - equivalently, species with distributions covering the greatest range of projection-induced area distortion.

**Fig. 3:**
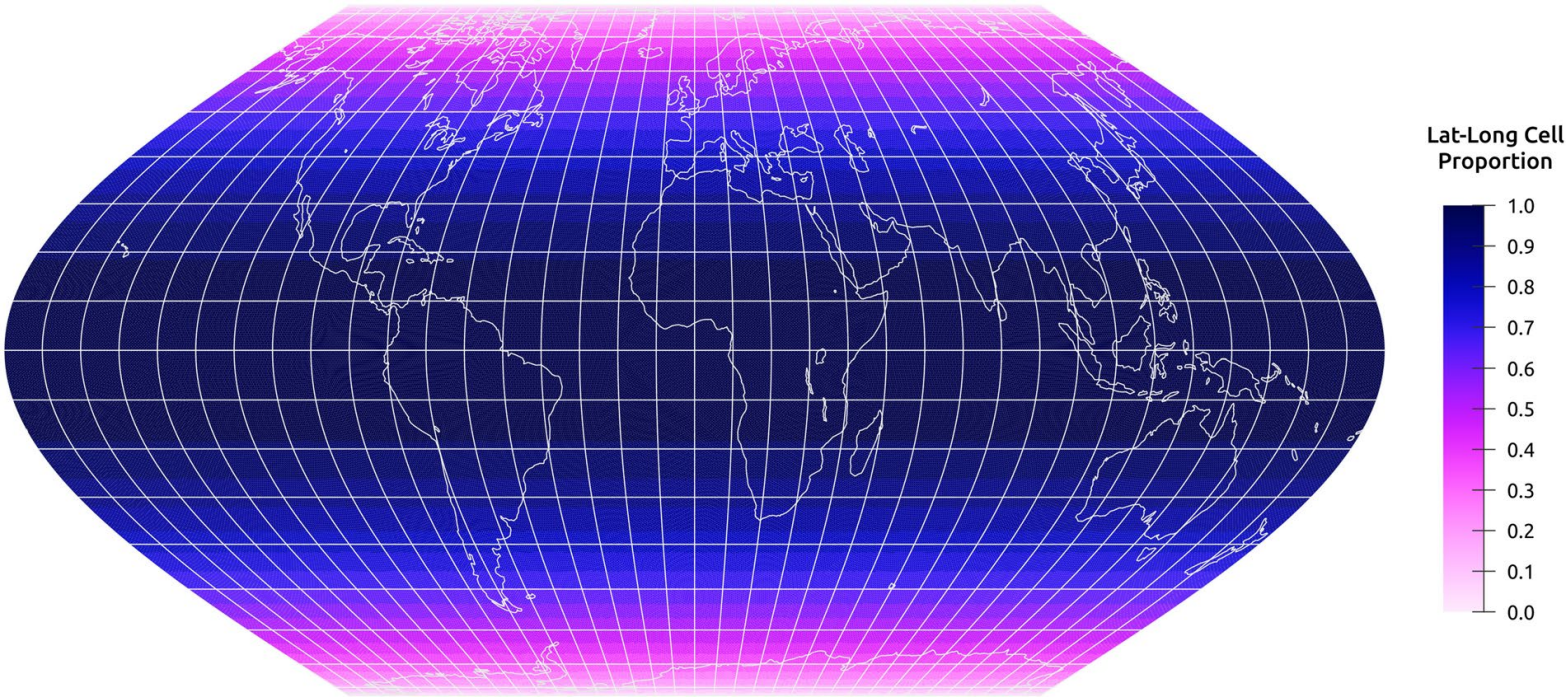
The area of raster pixels in the WGS84 coordinate reference system, expressed as a fraction of pixel area at the Equator. Fractional pixel area decreases with latitude to nearly 0.0 at ±90°, and serves as the measure of projection-induced area distortion used in this case study.

Area distortion was quantified as follows. First, working in the plate carrée map projection (in which WorldClim raster pixels are equal-area), a small circle centered at 0° latitude was drawn, and its geodesic area calculated using the GeographicLib library^54^. This represented the maximum possible geodesic area for a circle of this radius, in this projected coordinate reference system. Next, a circle of equal radius was drawn at each ISEA3H09 cell centroid, and its geodesic area calculated. The area of each of these circles, expressed as a fraction of the area of the 0° reference circle, served as the measure of area distortion with latitude assigned to each ISEA3H09 cell. These fractions, which vary from near 1.0 at the Equator (indicating low distortion) to near 0.0 at the poles (indicating high distortion), are mapped in Fig. 3.

Ranges from the IUCN Red List^50^ were used to characterize the geographic distributions of all species in the four mammalian orders Artiodactyla, Perissodactyla, Primates, and Proboscidea; species were defined as present in ISEA3H09 cells in which the species’ *fraction* attribute was greater than or equal to 0.5. For each species, a vector of the area distortion values of the ISEA3H09 cells in which it was present was compiled, and distortion percentiles, from 0 to 100 by 10, were calculated. Finally, species were sorted by the range between the 10^th^ and 90^th^ percentiles. The 10 species having the greatest 10^th^-90^th^ percentile ranges are listed in Table 13; these species were selected for comparison of bioclimatic envelopes derived from DGGS cells and raster pixels.

**Table 13:** The 10 large mammalian species with geographic distributions covering the greatest 10^th^-90^th^ percentile range of latitude-longitude raster pixel area distortion.

Binomial Name	Common Name	ISEA3H	Lat-Long Raster Pixel Proportions			
		Cell Count	Q10	Q50	Q90	Q10-Q90 Range
*Odocoileus virginianus*	White-Tailed Deer	5584	0.633	0.808	0.993	0.360
*Sus scrofa*	Wild Boar	10787	0.622	0.781	0.940	0.318
*Odocoileus hemionus*	Mule Deer	2443	0.562	0.733	0.856	0.294
*Lama guanicoe*	Guanaco	631	0.665	0.792	0.939	0.274
*Rangifer tarandus*	Reindeer	7160	0.332	0.466	0.599	0.268
*Capreolus capreolus*	Roe Deer	2466	0.487	0.652	0.752	0.264
*Capreolus pygargus*	Siberian Roe Deer	4617	0.543	0.645	0.806	0.263
*Alces alces*	Moose	8876	0.408	0.542	0.661	0.253
*Ovibos moschatus*	Muskox	625	0.172	0.330	0.425	0.253
*Ovis canadensis*	Bighorn Sheep	140	0.631	0.765	0.869	0.238

**Fig. 4:**
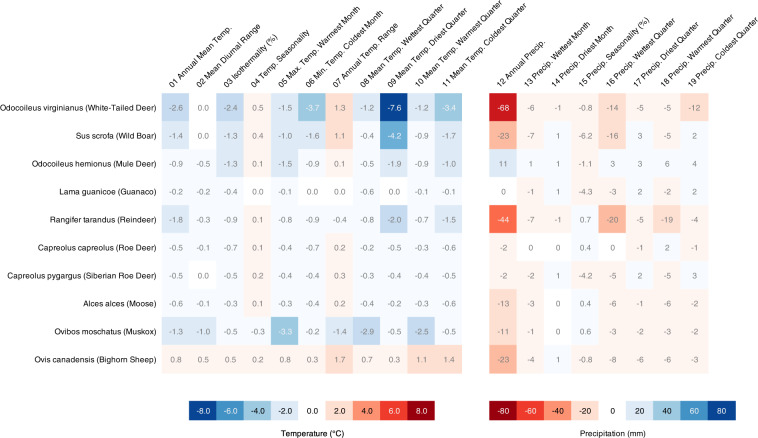
Differences in the median values of the 19 bioclimatic variables within each species’ geographic range, calculated via two approaches: the baseline, raster-based approach versus the ISEA3H DGGS approach.

For each selected species, bioclimatic conditions within the species’ range were characterized. The median values of the 19 bioclimatic variables (from WorldClim v2.0^31^) within each range were calculated using two different approaches. In the pixel-based, baseline approach, values of the WorldClim raster pixels within each species’ range were used to calculate bioclimatic medians (using the *raster* package^53^ for R). In the DGGS-based approach, *centroid* values of the ISEA3H09 cells in which each species was present were used to calculate bioclimatic medians. Differences between the two sets of medians, for temperature- and precipitation-related bioclimatic variables, are shown in Fig. 4. Niche distortion was quantified by subtracting ISEA3H09 medians from raster pixel medians; thus negative values indicate pixel-based medians are colder or drier, while positive values indicate pixel-based medians are warmer or wetter.

#### Results & discussion

All selected species are widely-distributed members of the order Artiodactyla, representing the families Cervidae (the white-tailed deer, mule deer, reindeer, roe deer, Siberian roe deer, and moose), Suidae (the wild boar), Camelidae (the guanaco), and Bovidae (the muskox and bighorn sheep).

The white-tailed deer (*Odocoileus virginianus*) exhibits the greatest 10^th^-90^th^ percentile distortion range; area distortion values range from 0.633 (indicating northernmost raster pixels within the species’ range have approximately two-thirds the area of pixels at the Equator), to 0.993 (indicating nearly no projection-induced area distortion in the southernmost portion of the species’ range). *O. virginianus* is distributed across North and Central America and northernmost South America, from southern Canada to Peru, absent only in the American Southwest. The species also exhibits the greatest shifts in median bioclimatic conditions; pixel-based absolute temperature estimates are uniformly lower, and temperature seasonality and range estimates uniformly higher, reflecting the over-representation of northern conditions. The pixel-based mean temperature of the driest quarter (BIO09) measures approximately 7.6 °C cooler.

This pattern of distortion in temperature-related bioclimatic variables is evident for the other species as well, with the exception of the bighorn sheep (*Ovis canadensis*). This species is present in a highly fragmented range across western North America, from the Columbia Mountains and Interior Plateau of Canada’s British Columbia, to the southern end of Mexico’s Baja California peninsula. The less than 1 °C differences observed in most temperature-related variables for the species likely result from edge effects, as ISEA3H09 cells are large relative to the species’ range fragments.

In principle, we expect environmental phenomena exhibiting a latitudinal gradient to suffer more from the biasing effect of projection-induced area distortion. Consider a phenomenon which is effectively random with respect to latitude; the over-representation of higher-latitude regions would not, in expectation, skew summary statistics in any systematic direction. Thus, we see more of an effect in temperature-related bioclimatic variables than in precipitation-related variables, as the latitudinal gradient in temperature is more pronounced: all but 12 of the 70 absolute differences in precipitation measure less than 10 millimeters.

While the difference of 2.6 °C in annual mean temperature (BIO01) observed over the range of *O. virginianus* may seem small, we note that a global increase of even 1.5 °C, the target of the Paris Agreement, is expected to have major impacts on species’ ranges^85^. These small but significant differences, primarily in temperature-related bioclimatic variables, demonstrate that the use of an equal-area grid may notably reduce niche distortion in species distribution and ecometric modeling, relative to the use of unequal latitude-longitude raster pixels.

### Eco-ISEA3H applications

Components of the Eco-ISEA3H database^37^ have been validated in several global macroecological studies. MCD12Q1 land cover classes and WorldClim bioclimatic variables were used to predict human activity, as evidenced by human-modified land cover, on the basis of present climatic conditions^86^. The PHYLACINE present-natural range of the Asian elephant (*Elephas maximus*), WorldClim bioclimatic variables, and CCSM4-based ETCCDI extremes indices were used to model global habitat suitability for *E. maximus* under present and future climatic conditions, to assess the species’ potential for conservation translocation^87^. Finally, biogeographic realms were used in a study of the sensitivity of ecometric estimates of present climate to the discovery of large, herbivorous mammal species over the past several centuries^88^. Results of these studies confirmed the biogeographic integrity of these component datasets; however, this is the first publication of the full Eco-ISEA3H database product and development methodology.

## Usage Notes

### ISEA3H resolutions

The Eco-ISEA3H database^37^ includes six consecutive, nested resolutions of the ISEA3H DGGS^34^, from resolution 5 to 10. Hexagonal grid cell areas range from approximately 210,000 square kilometers at resolution 5, to approximately 900 square kilometers at resolution 10; with each stepwise increase in resolution, average cell area is reduced to a third the value of the previous resolution. The average distance between grid cell centroids and first-order neighboring centroids ranges from approximately 450 kilometers at resolution 5, to 29 kilometers at resolution 10. Cell counts, areas, and spacings for each resolution are listed in Table 14.

**Table 14:** Grid specifications for ISEA3H resolutions 5 to 10.

Resolution	Cells	Area (*Km*^2^)	Centroid Spacing (*Km*)
5	2432	209903.5	452.5
6	7292	69967.8	261.2
7	21872	23322.6	150.8
8	65612	7774.2	87.1
9	196832	2591.4	50.3
10	590492	863.8	29.0

Most studies in which ecometric traits are prototyped^8,10^, or in which ecometric models are developed for environmental prediction^12,13,16,18,19^ or methodological comparison^89,90^, utilize a continental or global point grid with a nominal spacing of approximately 50 kilometers. Most of these derive from an equidistant point grid developed by Polly^8^, in which points were placed along equally-spaced latitudes, with longitudinal spacing scaled by the sine of the latitude. In addition, first-generation ecometric models utilized a grid with 0.5° latitude/longitude spacing^9,15^. While these grids maintain approximately 55.5 kilometer north-south spacing globally, east-west spacing varies with latitude, measuring approximately 55.5 kilometers at the Equator, 48.2 kilometers at ±30° latitude, and 27.9 kilometers at ±60° latitude.

To maintain consistency with previous ecometric model development, we recommend ISEA3H resolution 9 for ecometric studies utilizing the Eco-ISEA3H database^37^. At resolution 9, cell centroids are spaced approximately 50.3 kilometers. We note, however, that studies which examined the effect of grid resolution on ecometric model development found no significant differences in models built on 50-kilometer and coarser resolution grids: 50- and 75-kilometer spacing in a study of bovid locomotor traits in sub-Saharan Africa^12^; and 50-, 100-, and 250-kilometer spacing in a study of phenotypic, ecological, reproductive, and dietary traits in North American terrestrial mammals^89^. Thus coarser ISEA3H resolutions may be appropriate for ecometric studies in which computational complexity is a limiting factor.

### Point vs. area-integrated sampling

The Eco-ISEA3H database^37^ contains source datasets sampled and summarized spatially, via the global, hexagonal grids of the ISEA3H DGGS. The values assigned each ISEA3H cell represent either *point* samples or *area-integrated* summaries of the values of these source datasets. Specifically, the *centroid* attribute records the value of a source dataset at a single point, the centroid of each hexagonal cell. The *fraction*, *mode*, and *mean* attributes summarize spatial heterogeneity in a source dataset, within each hexagonal cell. For raster datasets, these latter statistics summarize multiple pixel values, and for vector datasets, the attributes and geometries of polygons within the spatial bounds of each cell.

As this suggests, area-integrated summaries require multiple observations within each ISEA3H cell. These statistics “see” the source dataset not at a single point, but at many: at each raster pixel, or across the region of the Cartesian plane bounded by the vector boundaries of a hexagonal cell. As the number of observations increases, these summaries tend toward integrals of continuous functions. Thus, it may be useful to note the number of raster pixels, for example, contained within each cell, for a source dataset and ISEA3H resolution of interest. Consider a 30 arc-second source raster dataset (for example, WorldClim v1.4^30^ and v2.0^31^), sampled at ISEA3H resolution 9. In this case, ISEA3H09 hexagonal cells contain a minimum of 2978 raster pixels, and a median of 3485 raster pixels.

Point and area-integrated sampling methods produce differing summary statistics. As a general rule, area-integrated summaries are more averaged than point samples, and provide results characteristic of each ISEA3H cell as a whole. Point samples are noisier than area-integrated summaries, but are more likely to retain extreme values or uncommon classes. The most appropriate method depends on the research question to be addressed; each provides a differing, but equally correct window on the source dataset.

#### Land cover classification: centroid vs. mode

While differences in the results of point and area-integrated sampling appear in both discrete and continuous source datasets, here we examine differences in land cover classification as reported by the *centroid* and *mode* attributes. Consider the MODIS land cover type (MCD12Q1) source dataset. Eco-ISEA3H hexagonal cells, at resolution 9, contain approximately 12,070 source raster pixels; each of these pixels is assigned one of 16 IGBP land cover classes, or a value indicating water cover. The difference in sampling methods may be quantified by the rate of correspondence between the land cover class reported by the two attributes. Again, the *centroid* attribute “sees” only a single raster pixel at each cell centroid, while the *mode* attribute sees all 12,070 pixels within each cell. Rates of correspondence between centroid- and mode-based land cover classification are reported globally and for each biogeographic realm, for the five years from 2014 to 2018, in Table 15.

**Table 15:** Percent of ISEA3H09 grid cells for which the MCD12Q1 land cover class, as represented by the *centroid* and *mode* attributes, correspond. Global and per-realm rates of correspondence are reported for the years 2014 to 2018.

Realm	Cell	Centroid vs. Mode Correspondence				
	Count	2014	2015	2016	2017	2018
Afrotropic	8312	81.7%	81.7%	81.6%	81.5%	81.4%
Antarctic	1312	96.8%	96.7%	96.8%	96.8%	97.0%
Australasia	3520	82.2%	82.2%	81.6%	82.2%	81.5%
Indo-Malay	3258	67.6%	67.3%	67.2%	66.7%	66.3%
Nearctic	7951	69.0%	68.8%	68.6%	68.5%	68.6%
Neotropic	7437	74.8%	74.5%	74.5%	74.4%	74.5%
None	4069	99.5%	99.5%	99.6%	99.6%	99.6%
Oceania	12	58.3%	58.3%	50.0%	50.0%	50.0%
Palearctic	20371	77.2%	77.1%	77.0%	77.2%	77.1%
Global	56242	78.2%	78.1%	78.0%	78.0%	77.9%

Globally, the two methods have a rate of correspondence of approximately 78%; thus, centroid- and mode-based land cover classification differs in approximately 22% of terrestrial ISEA3H09 cells. The highest rates of correspondence are recorded in the Antarctic realm, and in regions not assigned a realm by Olson *et al*.^56^, namely interior Greenland and Antarctica. Rates are higher than the global average in the Australasian and Afrotropic realms; lower than the global average in the Palearctic, Neotropic, Nearctic, and Indo-Malay realms; and lowest in Oceania, due to edge effects and small sample size.

**Fig. 5:**
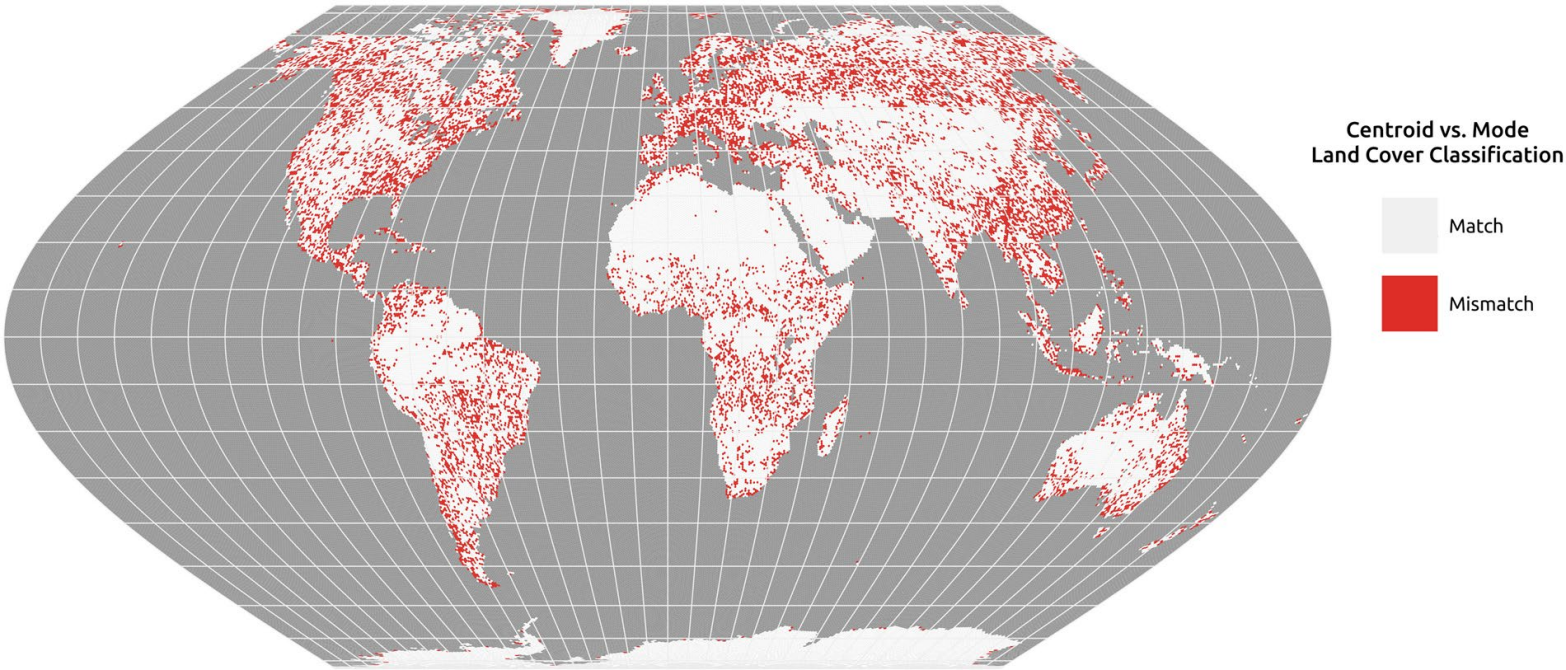
ISEA3H09 grid cells for which the 2018 MCD12Q1 land cover class, as determined by the *centroid* and *mode* attributes, correspond (white) or differ (red).

Centroid- and mode-based land cover correspondence is mapped in Fig. 5. The spatial pattern of matches and mismatches suggests rates of correspondence are greater in regions having largely uniform cover: the permanent snow and ice of Antarctica and Greenland, the barrens of the Sahara Desert and Arabian Peninsula, the evergreen broadleaf forests of the Amazon and Congo Basins, the open shrublands of central Australia, and the barrens and grasslands of central Asia. As landscapes become more varied, rates of correspondence decrease.

Mode-based classification results in more contiguous land cover patches, while centroid-based classification results in noisier cover, in which cells differ more often from neighbors. However, uncommon land cover classes are better represented in centroid-based classification. These uncommon classes rarely cover the greatest area within summarizing ISEA3H cells, but may happen to fall at ISEA3H cell centroids. For example, consider, among the natural land cover classes, the deciduous needleleaf forests (IGBP03), which occur only in northeast Asia. In the original MCD12Q1 dataset, these covered approximately 436,100 square kilometers in 2001. For ISEA3H09, this class was the mode value in just 32 cells, covering approximately 82,900 square kilometers (19% of the original); however, this class was the centroid value in 170 cells, covering approximately 440,500 square kilometers (101% of the original).

Similarly, among the human-modified land cover classes, consider urban and built-up lands (IGBP13), the global network of built, impervious surfaces constituting human settlements and infrastructure. In MCD12Q1, this class covered approximately 749,100 square kilometers in 2001. For ISEA3H09, this class was the mode value in 100 cells, covering approximately 259,200 square kilometers (35% of the original), and the centroid value in 282 cells, covering approximately 730,800 square kilometers (98% of the original). Thus, if representation of uncommon classes is important to the research question to be addressed, a point sample, as provided by the centroid attribute, may be more appropriate than an area-integrated summary.

### Vignette: predicting land cover classes

The tabular files of the Eco-ISEA3H database^37^ follow a relational model, in which the hexagon identification (HID) indexing number serves as primary key; at a given ISEA3H resolution, each cell is identified by a unique, sequential, integer HID, which may be used to link the records contained in any number of these tabular files. Use of the plain-text tabular files is straightforward, and will be illustrated in the following simple case study. Here, we will use R to predict natural land cover globally, based on bioclimatic variables, utilizing a decision tree approach to identify important climatic thresholds between land cover classes. Thus, this vignette offers a simplified version of the macroecological analysis presented by Beigaitė *et al*.^91^.

We begin by reading the MCD12Q1^69^ IGBP land cover classification for 2001; we use the first year for which these data are available, as we expect human modification of the landscape to be less extensive than in subsequent years. Here we read the tabular file containing the *mode* attribute, specifying the most common IGBP class within each ISEA3H cell - that is, the class covering the greatest area within each cell. Further, we use ISEA3H resolution 9, in which cells have an area of approximately 2,600 square kilometers, and a centroid spacing of approximately 50 kilometers. Note that tabular files have column headers, and are tab-delimited.

>igbp.m < - read.table(“ISEA3H09/MCD12Q1_V06/ISEA3H09_MCD12Q1_V06_Y2001_IGBP_Mode.txt”, header = TRUE, sep = “\t”)

We now have an R *data frame* containing a row for each ISEA3H09 cell, 196,832 rows in total. Note that an *IGBP_Mode* value of −1 indicates less than 20% of the cell’s area was covered by land (of all classes combined), and that values of 12, 13, and 14 indicate human-modified land cover classes, per the descriptions in Table 8. Next, we will subset the *data frame*, retaining only those cells in which a natural class predominates, covered at least 20% by land.

>igbp.m < - subset(igbp.m, IGBP_Mode %in% c(1:11, 15:16))

Following the subset, we retain 53,350 cells. However, defining terrestrial cells (and by extension, the spatial scope of our analysis) as those cells covered 20% or more by land is likely too inclusive. The *mode* attribute’s 20% threshold was implemented such that terrestrial datasets bounded by differing coastlines might still be layered together. The MCD12Q1 and WorldClim datasets, for example, are both clipped to the Earth’s terrestrial surface; however, each bounds that realm with its own coastline. Defining terrestrial ISEA3H cells as those covered 50% or more by one dataset (say, WorldClim) likely requires us to include cells covered less than 50% by the other (say, MCD12Q1).

The *mode* attribute’s 20% threshold allows for these differences around the edges; 20% is not intended to be a meaningful terrestrial/aquatic threshold in itself. Here, we will define terrestrial cells as those covered 50% or more by the MCD12Q1 dataset. Thus, we next read the tabular file containing the MCD12Q1 *fraction* attribute for 2001.

>igbp.f < - read.table(“ISEA3H09/MCD12Q1_V06/ISEA3H09_MCD12Q1_V06_Y2001_IGBP_Fractions.txt”, header = TRUE, sep = “\t”)

This creates a new *data frame*, containing the fraction of each ISEA3H cell’s area covered by each of the 16 IGBP land cover classes, from which the *mode* attribute we read previously was derived. For each cell, we sum these 16 fractions to find the total portion of the cell covered by land. We save this result to a new *Total* column.

>igbp.f$Total < - apply(igbp.f[, -1], 1, sum)

Finally, we subset the fractions *data frame* on the new *Total* column, retaining cells covered 50% or more by land (of all classes combined). The *summary* function allows us to verify the distribution of *Total* values after the subset.

>igbp.f < - subset(igbp.f, Total > = 0.5, select = c(HID, Total))

> summary(igbp.f$Total)


Min. 1st Qu. Median Mean 3rd Qu. Max.0.5001 0.9970 1.0000 0.9745 1.0000 1.0000


Following the subset, we retain 56,271 cells, having *Total* values between 50.01% and 100.00%. Now we merge the two *data frames*, containing MCD12Q1 *mode* and *fraction* attributes, using the HID as key. The merge function will return the intersection of the two sets: those ISEA3H cells retained in both *data frames* after the subsetting operation performed on each.

>igbp < - merge(igbp.m, igbp.f, by = “HID”)

Finally, we retain 51,047 cells, defining the spatial scope of our macroecological analysis; the new *data frame* contains both *mode* and *fraction* attributes for ISEA3H cells covered 50% or more by land, and with a natural land cover class predominating. The *mode* attribute serves as our label for each cell; next, we read the climatic variables we will use as predictors, the *mean* attribute for the 19 bioclimatic variables from WorldClim v2.0^31^.

>bio < - read.table(“ISEA3H09/WorldClim30AS_V02/ISEA3H09_WorldClim30AS_V02_BIO_Mean.txt”, header = TRUE, sep = “\t”)

We are ready now to assemble our global training dataset. We merge the two *data frames* containing labels and predictors, IGBP land cover classes and WorldClim bioclimatic variables.

>global.train < - merge(igbp, bio, by = “HID”)

Again, the *merge* function returns the intersection of the two sets; thus, our global training dataset contains 51,047 cells, each with a label (IGBP land cover class) and 19 predictors (WorldClim bioclimatic variables). Given the different coastlines used to bound the MCD12Q1 and WorldClim datasets, it is best practice to ensure no null values (−100) were retained in the bioclimatic variables; the *summary* function allows us to verify the distribution of mean annual temperature (BIO01) values in the training dataset.

>summary(global.train$BIO01_Mean)


Min. 1st Qu. Median Mean 3rd Qu. Max.-53.669 -2.420 15.156 8.577 24.215 32.498


The dataset is ready for use now in training a predictive model. Here we use a decision tree, as implemented in the *rpart* package^92^; we model the MCD12Q1 *mode* attribute (column 2 in our training *data frame*) as a function of the 19 WorldClim bioclimatic variables (columns 4 to 22). Note that we factor the *mode* attribute in the model formula, so that the *rpart* function fits a classification tree, rather than a regression tree on the integer IGBP land cover class codes.

>global.tree < - rpart(factor(IGBP_Mode) ~., data = global.train[, c(2, 4:22)])

This function returns an *rpart* object, containing, in this case, a fitted classification tree. This object may be passed to the *predict* function, along with the training dataset, to generate a vector of land cover predictions, one for each cell in the dataset. The *mode* attribute vector, and the vector of model predictions, may then be passed to the *table* function, to generate a confusion matrix.

>global.predict < - predict(global.tree, global.train, type = “vector”) 

> table(global.train$IGBP_Mode, global.predict)

**Fig. 6:**
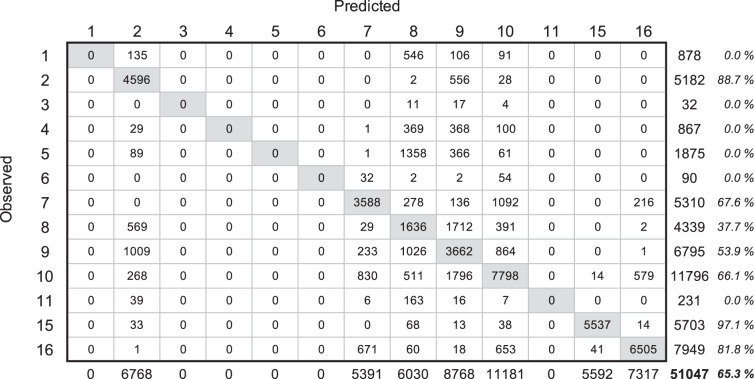
Confusion matrix for observed and predicted IGBP land cover.

The confusion matrix shown in Fig. 6 allows us to compare *observed* land cover, as defined by the MCD12Q1 *mode* attribute, and *predicted* land cover, as provided by our decision tree. The matrix summarizes both the distribution of predicted classes for each observed class, and inversely, the distribution of observed classes for each predicted class. These two perspectives on model performance reveal, for example, that our model has some difficulty in distinguishing between two tropical land cover classes: evergreen broadleaf forests (IGBP02) and savannas (IGBP09). Reading the second row of the matrix, we see that while 4,596 of 5,182 cells (88.7%) observed to be evergreen broadleaf forest were correctly classified, most cells of this class which were misclassified were classified as savanna (556 of 5,182 cells, 10.7%). Inversely, reading the second column of the matrix, we see that while 4,596 of 6,768 cells (67.9%) classified as evergreen broadleaf forest were observed to belong to this class, cells observed to be savanna (1,009 cells, 14.9%) constitute the largest misclassified land cover class.

**Fig. 7:**
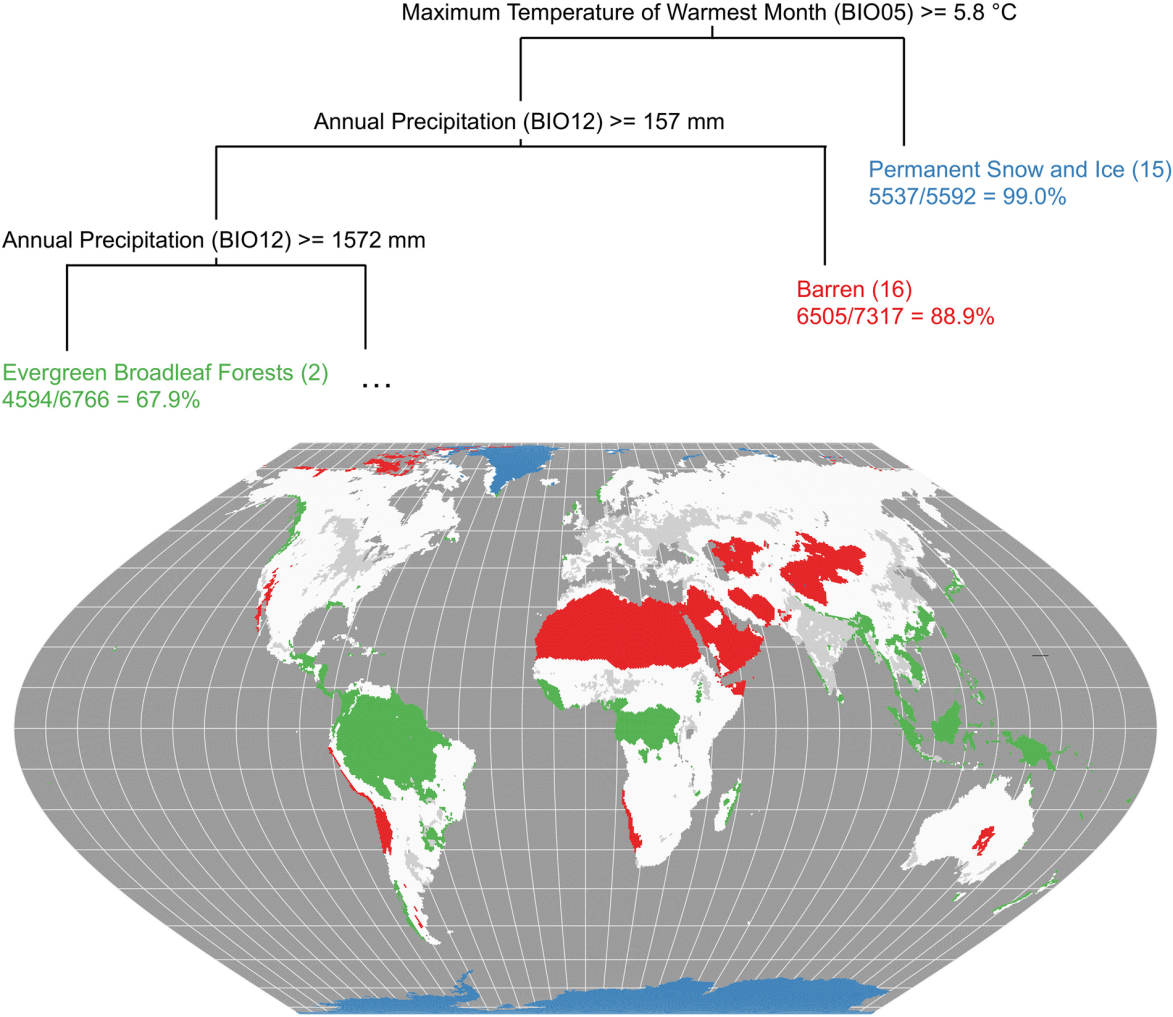
The first three splits of the IGBP land cover classification tree, and the spatial distribution of the predicted land cover classes.

Further, we can visualize the decision tree, and map the tree’s land cover classifications. The first three splits of the decision tree, and the spatial distribution of the predicted land cover classes, are shown in Fig. 7. At the tree’s root, maximum temperature of the warmest month (BIO05) below a very low threshold (approximately 6 °C) is used to identify permanent snow and ice (IGBP15), primarily in Antarctica and Greenland. For cells with a BIO05 value above the threshold, annual precipitation (BIO12) below a very low threshold (approximately 16 cm) is used to identify barren land (IGBP16), primarily in the Sahara, Arabian Peninsula, and central Asia. Finally, for cells with a BIO12 value above the threshold, BIO12 above a very high threshold (approximately 1.6 m) is used to identify evergreen broadleaf forests (IGBP02), primarily in the Amazon and Congo Basins and the Malay Archipelago. Areas remaining to be classified by further splits are shown in white, and areas excluded due to predominantly human-modified land cover are shown in gray.

## Data Availability

R and Python code developed for the Eco-ISEA3H database^37^ was committed to a public GitHub repository, and may be accessed via the following URL: https://github.com/mechenich/eco-isea3h.
